# Time-Resolved Native Mass Spectrometry for Direct Measurement of Biomolecular Kinetics

**DOI:** 10.1021/jacs.5c21842

**Published:** 2026-05-09

**Authors:** Virginia K. James, Lauren Stover, Hanieh Bahramimoghaddam, Tanishq Khandelwal, Jing-Yuan Chang, James Downing, Elena Scott, Kathleen O. Bailey, David H. Russell, Lane A. Baker, Arthur Laganowsky

**Affiliations:** Department of Chemistry, 14736 Texas A&M University, College Station, Texas 77843, United States

## Abstract

The functional outcomes of biomolecular interactions depend on the kinetics of association and dissociation between proteins and their binding partners, ranging from small molecules to other proteins, and are fundamental to understanding cooperativity, allostery, and drug action. However, existing kinetic methods, such as surface plasmon resonance and biolayer interferometry (BLI), require immobilization or labeling of one binding partner and are often indirect measurements. Here, we introduce a transformative time-resolved native mass spectrometry (MS) approach that enables direct, label-free, and immobilization-free quantification of biomolecular kinetics across diverse interactions within minutes using only picomolar sample amounts. We benchmarked the approach using well-characterized systems and obtained kinetic parameters that agreed with those measured by BLI. We further demonstrate the utility of time-resolved native MS in quantifying the kinetics of protein–small-molecule interactions, including those involving an irreversible inhibitor. By capturing the association and dissociation of biomolecular interactions in real time, time-resolved native MS overcomes longstanding limitations of conventional kinetic assays and transforms native MS from a static technique to a dynamic, quantitative tool for probing biomolecular kinetics and mechanisms that underpin therapeutic discovery.

## Graphical abstract

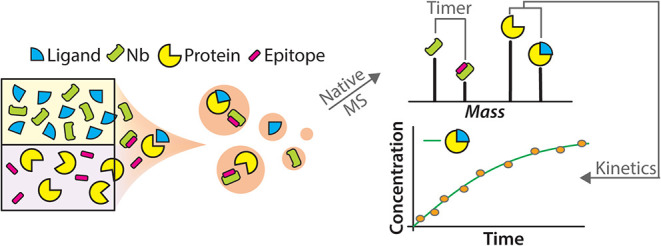

## Introduction

Interactions between proteins and small molecules, ions, nucleic acids, or other biomolecules are fundamental to nearly all biological processes and are governed by kinetic principles. The rate at which a ligand binds is defined by the association rate constant (*k*_on_), while the lifetime of the interaction is determined by the dissociation rate constant (*k*_off_).^1–3^ The association rates for noncovalent biomolecular interactions are typically on the order of 10^5^–10^6^ M^–1^ s^–1^ with an upper diffusion limit of 10^9^ M^–1^ s^–1^.^4,5^ This corresponds to interaction time scales on the order of microseconds to milliseconds, necessitating biophysical techniques capable of resolving such rapid binding events. Kinetics provides mechanistic insight into how binding at one site influences other interactions, revealing mechanisms of cooperativity and allosteric regulation.^6–8^ While thermodynamics defines the strength of molecular interactions,^9^ kinetics captures the conformational dynamics of macromolecules that influence both ligand binding and release.^10^ Dysregulation of these kinetic parameters underlies many diseases, including cases where pathological ligands exhibit prolonged binding or essential interactions dissociate too rapidly.^11^ Moreover, recent studies have shown that the *in vivo* efficacy of many drugs correlates more strongly with their kinetic properties than with their equilibrium thermodynamic parameters.^1,10,12^

A number of techniques have been developed to measure the kinetics of biomolecular interactions, such as surface plasmon resonance (SPR), biolayer interferometry (BLI), radioligand binding, time-resolved fluorescence resonance energy transfer, and nuclear magnetic resonance (NMR).^13,14^ These techniques can be broadly categorized into two groups: direct and indirect methods.^13^ Direct methods measure the actual concentrations of bound and free proteins, while indirect methods infer these concentrations based on an observed signal. NMR is a direct method that can provide thermodynamic and kinetic information on biomolecular interactions.^15^ In the case of NMR, a drawback is the need to incorporate stable isotopes and the requirement of high-concentration samples.^15,16^ Some labeling methods, such as fluorescence or radioisotope-based approaches, can quantify the concentration of ligands bound to proteins. However, the disadvantage is the modification of ligands with non-natural fluorophores or the incorporation of radionuclides.^17^ SPR and BLI are popular, indirect optical techniques that monitor changes in refractive index (SPR) or interference patterns (BLI) that are associated with the binding partner binding the immobilized ligand on the sensor surface. These methods enable the measurement of real-time binding and kinetic information. However, a limitation is that the protein (or ligand) requires immobilization on the sensor surface, which can alter the properties of the protein and its interaction with the target molecule.^14^ While these techniques have provided valuable insights into the kinetics of some biomolecular interactions, they are often time-consuming, require labeling and/or immobilization, and lack the potential of resolving the kinetics of multimeric protein systems.

Native mass spectrometry (MS) is a biophysical technique well-suited for the characterization of proteins and their interaction with other molecules.^18–20^ Unlike other techniques that report on the ensemble, native MS is a direct method that can capture a snapshot of solution equilibria and resolve individual ligand-bound states of protein complexes.^21,22^ While the technique has been used to determine equilibrium binding constants and thermodynamics,^23–28^ the use of native MS to determine kinetics of protein–ligand interactions has largely been limited to slow processes (for review see refs 20,29 ). Attempts to capture faster time scales using mass spectrometry have been made using specialized electrospray ionization techniques, such as extractive electrospray,^30,31^ fused droplet-electrospray ionization,^32,33^ multiple channel electrospray,^34,35^ dual-sprayer microchips,^36,37^ and liquid sampling desorption electrospray ionization.^38^ In particular, theta emitters feature a glass inner divider separating two channels, allowing controlled, rapid mixing of solutions within a Taylor cone^39^ common to both channels and by droplet fusion or coalescence^40^ during electrospray ionization (ESI).^41^ This is unlike traditional emitters that have one channel where equilibrium is reached prior to introduction of the sample into the mass spectrometer (Figure S1). Previous studies using θ emitters have demonstrated rapid mixing for noncovalent complexation formation of simple, small-molecule systems,^41,42^ (un)­folding and supercharging of proteins,^43–49^ reduction–oxidation reactions,^42^ and hydrogen/deuterium exchange reactions.^41,50^ Herein, we present a time-resolved native MS platform to quantify the kinetics of biomolecular interactions, including protein–protein and protein–drug interactions, under native conditions and without labels.

## Results

### Overview of the Time-Resolved Native MS Approach

As theta emitters have demonstrated success in capturing intermediates for simple chemical reactions, we hypothesized that theta emitters could be leveraged to determine biomolecular kinetics for protein–protein and protein–ligand interactions. However, realization of this goal requires overcoming a key limitation in estimating mixing times within theta emitters, which previously relied on estimates or simple chemical reactions that are not applicable to protein–ligand interactions. We therefore envisioned a solution to determine mixing times for theta emitters could be achieved through the incorporation of an internal standard (Figure 1), a concept widely adopted in quantitative analysis. The binding kinetics of an internal standard, such as a nanobody–epitope interaction, serve as a built-in timer for calibrating mixing times and can be implemented using one of two strategies (Figures 1A and S2). First, as most biochemical experiments utilize recombinant proteins containing affinity tags, the protein of interest can be expressed with an affinity or epitope tag recognized by a specific antibody or nanobody (Nb) (Figure S2A), constituting a “fusion” strategy. Alternatively, the epitope and its corresponding binding partner can be introduced into the same experiment as the unmodified protein of interest (Figure 1A), providing means for label-free kinetic measurements through a “non-fusion” strategy. Regardless of the chosen strategy, one channel of the theta emitter contains the protein of interest with its epitope tag (either fused or added separately), while the other channel contains the Nb that specifically recognizes the epitope together with the ligand that binds the protein of interest (Figure 1A). Application of voltage to initiate electrospray ionization enables the two channels to rapidly mix. The different Nb and ligand-bound states of the protein fused to the epitope tag can readily be resolved in the native mass spectrum (Figure 1B). To calibrate time (Figure 1C), the known binding kinetics for the epitope–Nb interaction can be used to generate the theoretical abundance of the complex as a function of time, given the starting concentration of the components, serving as a mixing timer. Notably, the differing affinities and kinetic properties of epitope–Nb interactions, including other timer pairs, define the optimal time window for accurately resolving mixing times (Figure S3). Based on this calibration, the fractional abundance of the protein-epitope bound to Nb (calculated from the native mass spectrum) can directly be used to determine the experimental mixing time. This mixing time, combined with the fractional abundance of the protein bound to ligand, provides a data point in the kinetic plot (Figure 1D). The overall procedure is repeated to generate a time-resolved native MS kinetic plot of the protein–ligand abundance at different mixing times (Figure 1E), which can be used to determine kinetic parameters (Figure 1F,G).

**Figure 1:**
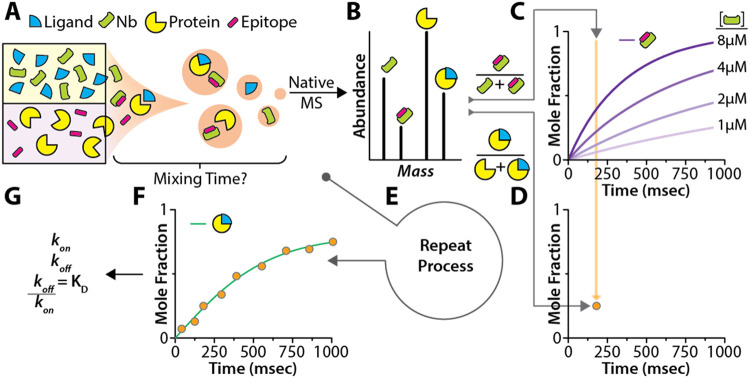
Overview of the time-resolved native mass spectrometry approach and determination of biomolecular kinetics. (A) One channel contains the protein of interest with an epitope tag, added as a nonfusion timer in isolation from the protein of interest, while the second channel contains the corresponding nanobody and ligand. Rapid mixing occurs at the emitter tip prior to ionization and transfer into the mass spectrometer. (B–G) Workflow of the time-resolved native MS approach, where the Nb–epitope interaction serves as a fusion timer to calibrate mixing times for each mass spectrum. (B) The native mass spectrum is deconvoluted and the abundance of the timer species is compared to (C) theoretical time-dependent binding curves to obtain a droplet lifetime for each spectrum. The curves shown in (C) represent an interaction with 1 μM protein containing an epitope binding to different initial concentrations of Nb based on a *k*_on_ of 3.6 × 10^5^ (M^–1^ s^–1^) and *k*_off_ of 9.4 × 10^–6^ (s^–1^). (D) The droplet lifetime for the spectrum is plotted against the abundance of ligand-bound species. (E) The process is repeated for numerous spectra until (F) there are enough points to generate a curve to fit for (G) kinetic parameters, including *k*_on_, *k*_off_, and *K*_D_. A schematic for a fusion timer experiment where the epitope is fused to the protein of interest is shown in Figure S2.

### Optimization and Benchmarking of the Time-Resolved Native MS Method

We next set out to establish simple 1:1 protein–protein systems to optimize and benchmark our method. To evaluate the fusion strategy, we expressed and purified the green fluorescent protein (GFP) containing a genetically encoded C-terminal α-synuclein (AS) epitope tag. This tag contains the four amino acid sequence (EPEA) that is specifically recognized by the anti-AS Nb (NbAS), which binds with a reported dissociation constant of approximately 100 nM.^51^ We also prepared nanobody (Nb15), a high-affinity anti-GFP binder with a reported dissociation constant in the single-digit picomolar range.^52^ The mass spectrum of a mixture containing both Nbs and GFP at equilibrium revealed a predominant signal corresponding to the fully bound ternary complex, along with low-abundant signals for the GFP–NbAS and GFP–Nb15 complexes (Figure 2A). In this model system, the GFP–NbAS interaction serves as an internal mixing timer, allowing the determination of the kinetics for the GFP–Nb15 interaction.

**Figure 2:**
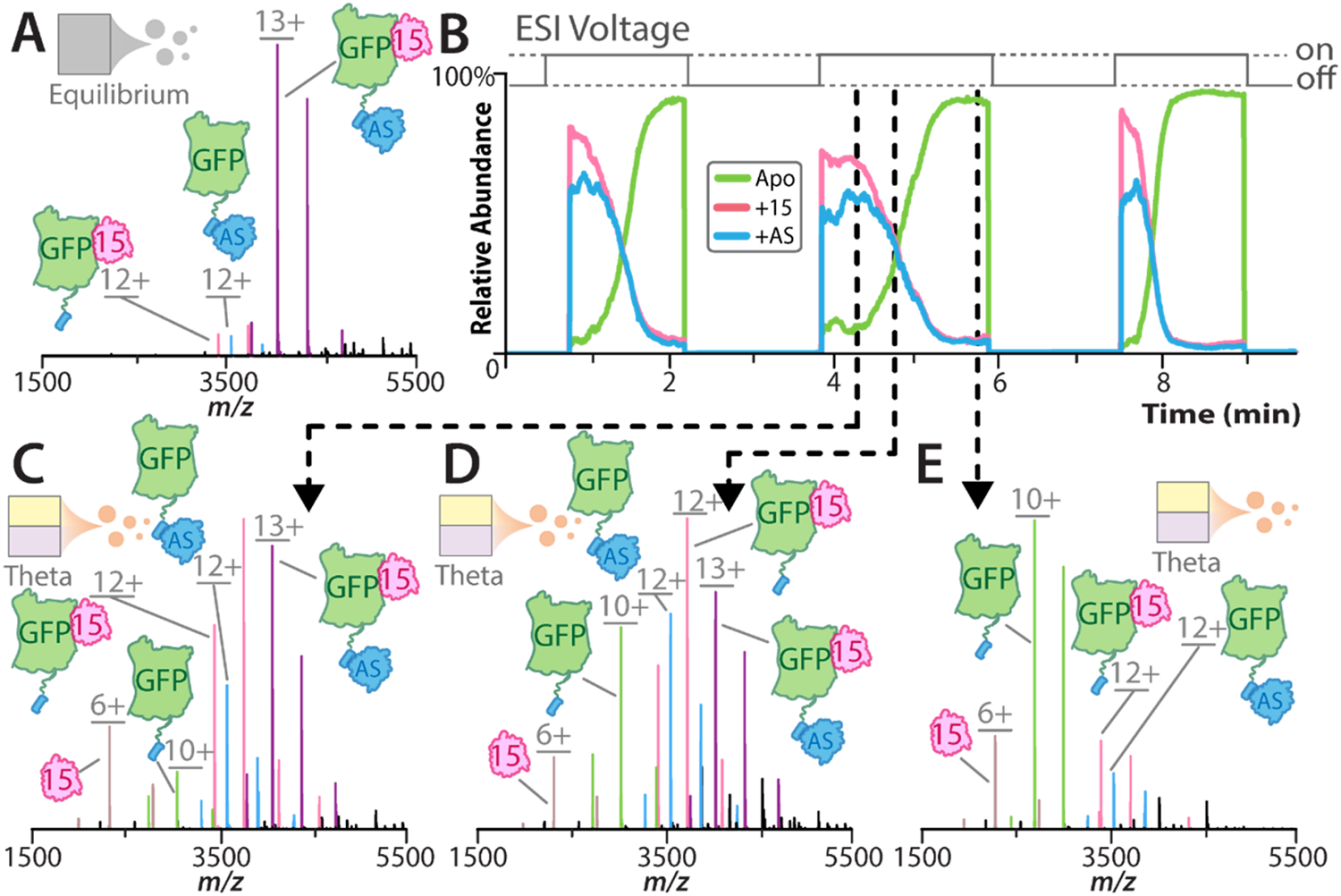
Capturing an array of mixing times using theta emitters. (A) Mass spectrum of 0.8 μM green fluorescent protein (GFP) binding 2 μM Nb15 (15) and 4 μM NbAS (AS) at equilibrium. (B) Extracted mass chromatogram of apo, Nb15, and NbAS-bound GFP over three ESI power cycles acquired in one data acquisition. (C–E) Mass spectra 0.8 μM GFP binding 2 μM Nb15 and 4 μM NbAS in a theta emitter shortly after ESI voltage was turned on (C), and after (D) 1 or (E) 1.5 min of sustained voltage. The concentrations listed account for a 2-fold dilution within the Taylor cone during rapid mixing. Deconvoluted spectra are shown in Figure S9.

For the nonfusion strategy, we developed two timer systems, Son of Sevenless catalytic domain (SOS^cat^)^53^ and GFP genetically encoding a C-terminal ALFA tag, an epitope that binds to its corresponding nanobody with low picomolar affinity (NbALFA).^54^ The ALFA tag is particularly well-suited for timing applications due to its slow dissociation rate,^54^ ensuring that the nanobody remains bound over the time scale of time-resolved native MS. These ALFA-tagged constructs were used to time the GFP–Nb15 and GFP–NbAS interactions. At equilibrium, both constructs were nearly fully bound to NbALFA alongside the nanobody complexes they were designed to time (Figures S4 and S5). Together, these three systems enable cross-validation of time-resolved native MS across distinct protein–protein interactions and allow direct comparison of the fusion and nonfusion timer strategies.

Mixing times of θ emitters have been reported to be influenced by parameters such as tip diameter, backing pressure, and distance of emitter to orifice,^46,50,55–57^ prompting us to optimize conditions to capture binding intermediates using θ emitters. Similar to previous results, we found that varying these parameters resulted in different abundances of immediate states (Figure S6). However, these conditions alone were not sufficient to generate the broad dynamic range of mixing times to accurately determine protein–ligand kinetics. While evaluating known parameters that influence mixing times, we discovered that the abundance of the GFP–Nb15 and GFP–NbAS complexes was dependent on the duration for which the ESI voltage was applied (Figure 2B). At the onset of ESI voltage application in θ-emitter experiments, we observed a high relative abundance of the GFP–NbAS complex (Figure 2C). However, as data acquisition continued with sustained ESI voltage, the abundance of this complex gradually decreased, accompanied by an increase in the signal corresponding to free GFP (Figure 2D,E). This time-dependent shift in complex distribution indicates that mixing times are greater when spraying voltage is initially applied, likely due to partial premixing of the two solutions at the tip of the theta emitter before stable spray formation. Indeed, by repeatedly toggling the ESI voltage on and off, we collected bursts of native mass spectra that captured varying abundances of complex formations (Figure 2B). Similar results were found for SOS^cat^ ALFA and GFP ALFA interacting with NbALFA (Figures S7A–E and S8A–E, respectively), demonstrating that the method is reproducible across different systems.

Beyond ESI power cycles, we found that emitter diameter must be sufficiently large (∼20 μm) to promote mixing and prevent emitter clogging (Figure S10). While emitters of larger diameters can lead to decreased sensitivity, especially for larger protein complexes, we varied emitter diameter while monitoring the ratio of apo GFP to GFP–Nb15 complex and found no distinguishable differences across emitter diameters (Figure S11). In short, the optimized conditions enable the detection of reaction intermediates over a broad temporal range, with a wide range of mixing times captured in as little as a 2 min data acquisition.

To benchmark the method, we measured the kinetics of all three model systems and their timers using biolayer interferometry (BLI), an established method for quantifying biomolecular binding kinetics. We first obtained kinetics using BLI under the same buffer conditions used for native MS for both ALFA systems (SOS^cat^ and GFP). Interestingly, the two systems had significantly different association rates (*k*_on_ of 1.931 ± 0.002 × 10^5^ M^–1^ s^–1^ and 2.256 ± 0.003 × 10^4^ M^–1^ s^–1^) and, therefore, affinities (*K*_D_ of 234.7 ± 0.5 pM and 1.379 ± 0.007 nM) (Figures S12–13, for SOS^cat^ and GFP, respectively), despite containing the same C-terminal epitope. We then determined the kinetics of the GFP–NbAS interaction, obtaining a *k*_on_ of 1.17 ± 0.01 × 10^5^ M^–1^ s^–1^ and *k*_off_ of 1.163 ± 0.003 × 10^–1^ s^–1^, corresponding to a *K*_D_ of 1.00 ± 0.01 μM (Figure 3A). Similar experiments were performed for Nb15 interacting with GFP, yielding a *k*_on_ of 8.6 ± 0.3 × 10^4^ M^–1^ s^–1^, *k*_off_ of 1.41 ± 0.01 × 10^–3^ s^–1^, and *K*_D_ of 16.3 ± 0.5 nM (Figure 3B). In parallel, we also determined the crystal structure of the GFP–Nb15 complex, revealing a buried surface area of 635 Å^2^ for the noncovalent interaction (Figures 3C and S14 and Table S1). Nb15 binds to the side of the GFP β barrel centered around Lys115, opposite the C-terminus containing the EPEA tag. This spatial separation indicates that NbAS and Nb15 can simultaneously bind GFP, rendering the system well-suited for the fusion timer strategy.

**Figure 3:**
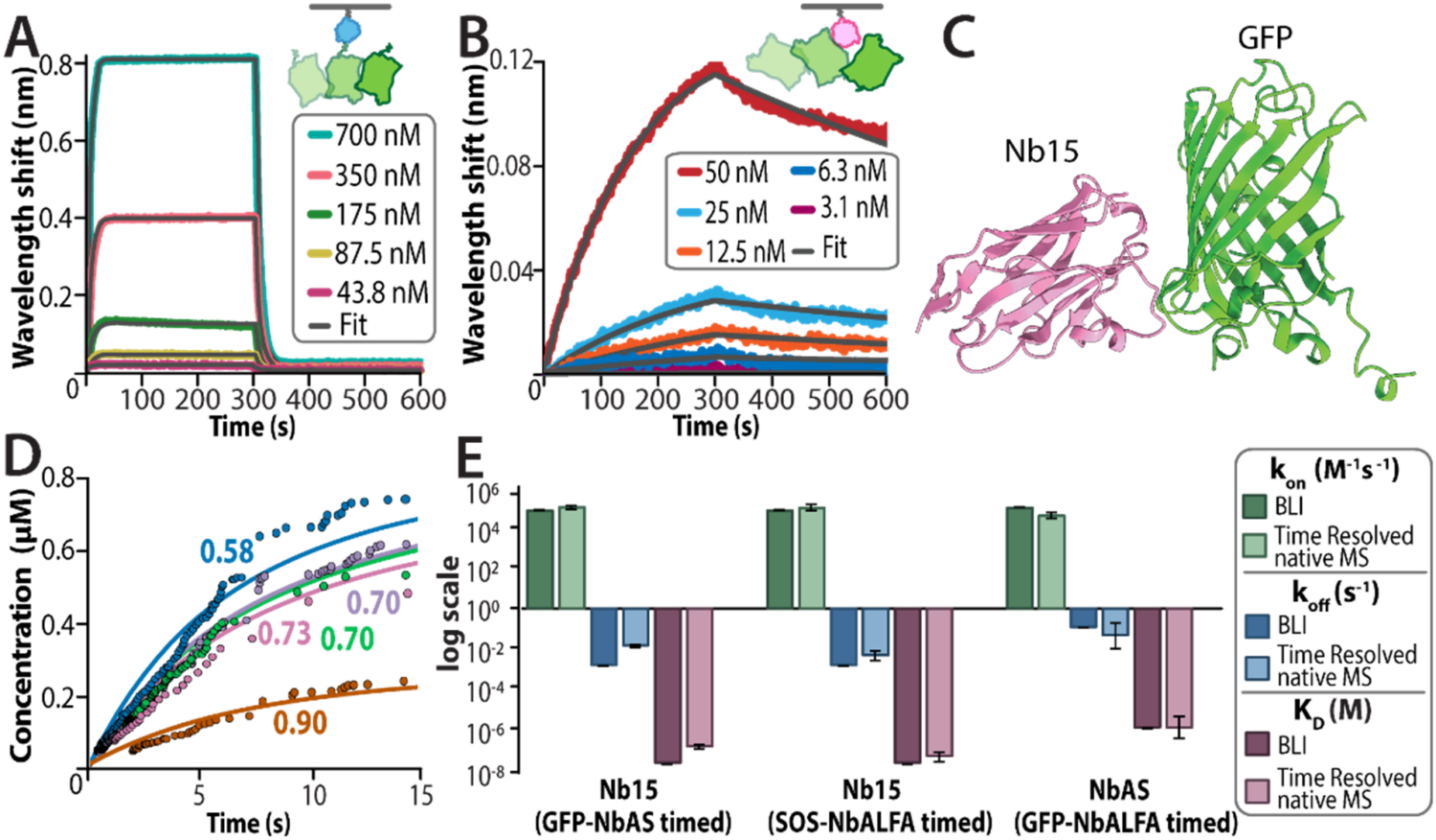
Capturing kinetics of protein–protein interactions using theta emitters. (A, B) Biolayer interferometry (BLI) sensorgrams showing GFP binding to biotinylated (A) NbAS and (B) Nb15, with corresponding global fits (solid lines) based on a 1:1 binding model. (C) Crystal structure of Nb15 bound to GFP (Table S1 and Figure S14). (D) Time-resolved native MS kinetic curves showing GFP binding to Nb15, timed using NbAS. Data from five bursts are plotted in distinct colors, fit to a 1:1 global kinetic model in which each burst is assigned its own mixing factor (α). The colored traces represent individual bursts, and solid lines denote the corresponding model fits, with each α value labeled in the matching color. (E) Kinetic rate constants and equilibrium dissociation constants (*K*_D_) for the GFP–Nb15 interaction timed with GFP-NbALFA and SOS–NbALFA, and for the GFP–NbAS interaction timed with GFP-NbALFA, as determined by BLI and time-resolved native mass spectrometry. A table of kinetic values for each system is provided in Table S2.

We next determined the kinetics of all three model systems by time-resolved native MS and compared the results to those obtained by BLI. For the interaction of GFP with Nb15 and NbAS, we initially computed the expected binding curve for the GFP–NbAS complex using the experimental kinetic parameters determined by BLI and the final concentration predicted after rapid mixing in theta emitters, corresponding to an expected 2-fold dilution of the starting components. This calibration curve was used to determine the mixing time for each mass spectrum in each burst, initiated by the application of ESI voltage. Assuming equal mixing, the abundance of GFP–NbAS in the bursts indicated that mixing times varied from 0 to 40 s (Figure S15). Upon closer inspection, we observed that, in some cases, individual bursts of data deviated markedly from the fitted kinetic curve, yielding distinct binding profiles in which the relative abundance of the GFP–Nb15 complex differed from that of GFP–NbAS at a given mixing time (Figure S16A). We surmised that these discrepancies likely arose from unequal mixing or deviations from the expected 2-fold dilution of the chamber components, possibly due to asymmetries in the θ emitter or uneven channel flow/openings (Figure S10).

To account for unequal mixing, we incorporated a mixing factor (α) into our kinetic fitting routine to correct for deviations in the dilution of components in the two chambers. For an ideal 2-fold dilution after rapid mixing, α equals 0.5, whereas uneven mixing (α ≠ 0.5) alters the calibration curve and, consequently, the determined mixing time (Figure S17). When globally fitting data from multiple bursts (ranging from 3 to 10) collected using the same emitter while allowing a single mixing factor to vary, we observed improved agreement between the experimental data and the fitted kinetic curve (Figure S16B). Allowing the mixing factor to vary for each burst further enhanced the fits and was statistically justified based on model comparison criteria (Figure 3D). Using this approach, we determined the kinetics of the GFP–Nb15 interaction, obtaining a *k*_on_ of 1.3 ± 0.3 × 10^5^ M^–1^ s^–1^ and *k*_off_ of 1.3 ± 0.2 × 10^–2^ s^–1^, corresponding to a *K*_D_ of 110 ± 30 nM using the fusion strategy. For the nonfusion approach, we choose to use the ALFA system as a timer, given that its dissociation rate is likely not measurable on the time scale of time-resolved native MS for both systems (Figures S12–13). Using SOS^cat^ ALFA-NbALFA as a nonfusion timer, we determined kinetics for the GFP–Nb15 interaction with the same burst variable mixing factor fitting regime (Figure S18). We obtained a *k*_on_ of 1.2 ± 0.4 × 10^5^ M^–1^ s^–1^ and *k*_off_ of 5 ± 3 × 10^–3^ s^–1^, corresponding to a *K*_D_ of 40 ± 20 nM, in close agreement with the fusion timed GFP–Nb15 results. The GFP ALFA-NbALFA system was used to determine the kinetics of the GFP EPEA-NbAS interaction, which was also fit with a burst variable mixing factor (Figure S19) and yielded a *k*_on_ of 5 ± 2 × 10^4^ M^–1^ s^–1^ and *k*_off_ of 1 ± 2 × 10^–1^ s^–1^, corresponding to a *K*_D_ around 2 ± 2 nM. Furthermore, all three systems are in close agreement with those obtained from BLI (Figure 3E and Table S2). These results demonstrate that the time-resolved native MS approach can quantitatively determine biomolecular kinetic parameters consistent with those derived from traditional indirect methods.

As prior work has shown differences in MS response factors between protein–protein and protein–ligand complexes, which have been shown to affect calculated affinities^58,59^ and thus may affect kinetics determined by time-resolved native MS, we also evaluated whether response factors differed between the apo protein and protein–Nb complex. We varied the ratio of apo GFP ALFA to purified GFP ALFA-NbALFA complex, which should have no discernible dissociation rate in our experiments. We found that the abundances of apo GFP ALFA and GFP ALFA-Nb complex determined with native MS differed slightly from solution concentrations determined by absorbance measurements (Figure S20). However, accounting for this slight difference in concentration resulted in no statistical difference in the measured kinetics (Figure S21 and Table S2).

### Quantifying Noncovalent and Irreversible Protein–Drug Binding Kinetics

After benchmarking the time-resolved native MS method, we next investigated whether the approach could be applied to determine the kinetics of protein–drug interactions. To this end, we selected monomeric proteins that have well-characterized interactions with small-molecule ligands. For these experiments, we chose the label-free approach, in which GFP and Nb15 are added to separate chambers to function as an internal mixing timer. First, we examined the noncovalent interaction between carbonic anhydrase II (CA), an enzyme that catalyzes the formation of bicarbonate, and ethoxzolamide (Eth), a drug developed for the treatment of glaucoma.^60^ At equilibrium, CA was mostly Eth-bound and GFP was bound to Nb15, while in a theta emitter, apo, drug-bound, and Nb15-bound states for CA and GFP were detected (Figure 4A). Using the time-resolved native MS method, we determined the binding kinetics of CA–Eth interaction, obtaining a *k*_on_ of 1.7 ± 0.9 × 10^6^ M^–1^ s^–1^ and a *k*_off_ of 6 ± 3 × 10^–2^ s^–1^, corresponding to a *K*_D_ of 40 ± 10 nM (Figure 4B). These values are in close agreement with previously reported values, with *K*_D_ ranging from 2 to 24 nM, determined in different buffer systems.^61,62^ Similar results were found at equilibrium and in theta experiments when examining the noncovalent interaction between dihydrofolate reductase (DHFR), an enzyme that reduces dihydrofolate to tetrahydrofolate, and methotrexate, a drug used in the treatment of some cancers and autoimmune diseases (Figure 4C).^63^ For this interaction, we determined a *k*_on_ of 1.3 ± 0.9 × 10^6^ M^–1^ s^–1^ and *k*_off_ of 3 ± 2 × 10^–3^ s^–1^ (Figure 4D), corresponding to a *K*_D_ of 2 ± 1 nM, which is in excellent agreement with previously reported literature values.^64,65^ Collectively, these findings confirm that time-resolved native MS can reliably quantify the kinetics of reversible protein–drug interactions in close agreement with established methods.

**Figure 4:**
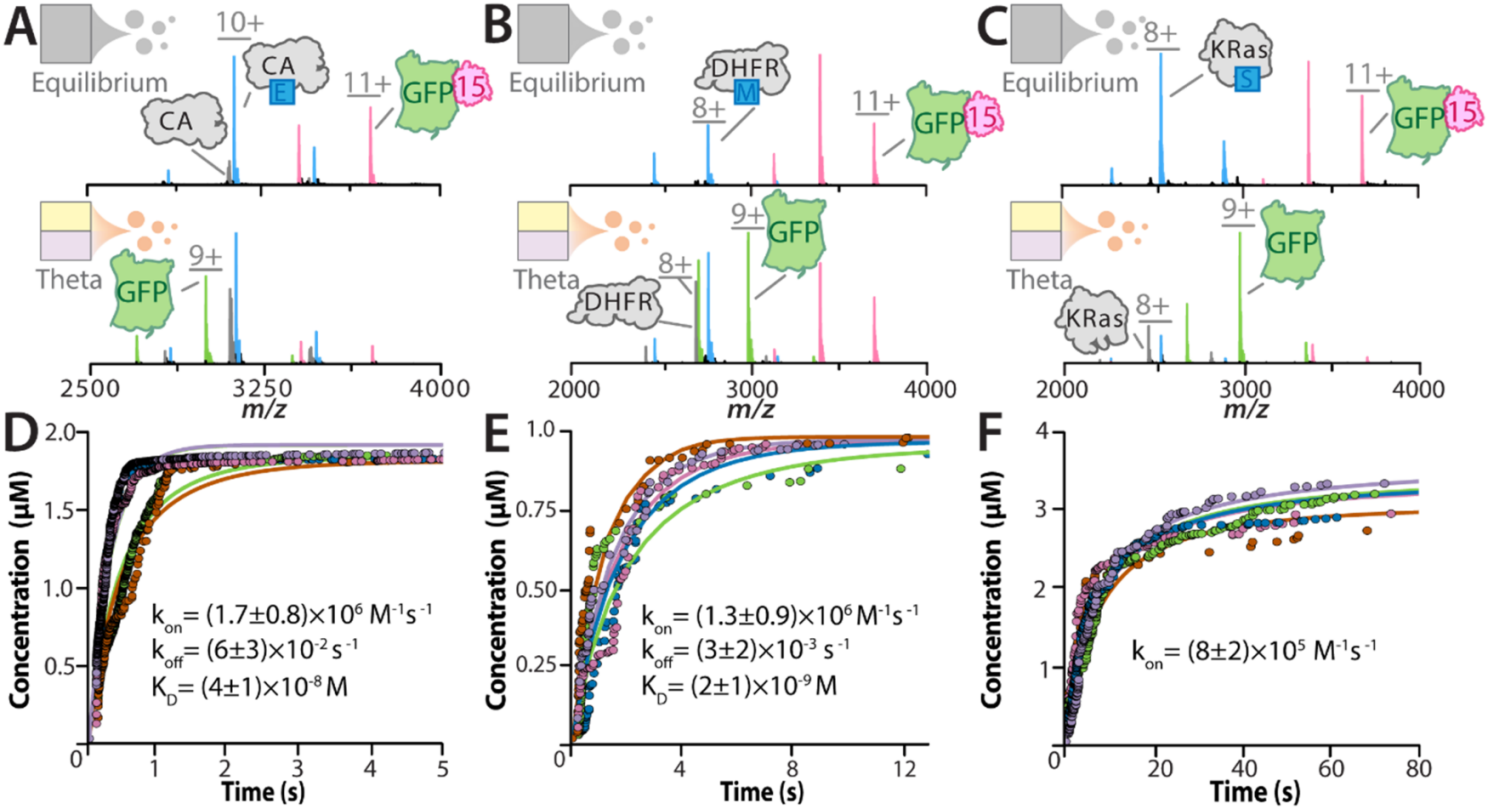
Determination of protein–ligand kinetics using time-resolved native MS. (A) Mass spectra of a solution containing 2 μM carbonic anhydrase (CA), 0.4 μM green fluorescent protein (GFP), 2 μM ethoxzolamide (E), and 2 μM Nb15 (15) at equilibrium (top panel) and in a theta emitter (bottom panel). (B) Time-resolved native MS kinetic curves for CA binding ethoxzolamide. (C) Mass spectra of 1 μM dihydrofolate reductase (DHFR), 0.5 μM GFP, 1 μM methotrexate (M), and 1 μM Nb15 at equilibrium (top panel) and in a theta emitter (bottom panel). (D) Time-resolved native MS kinetic curves for DHFR binding methotrexate. (E) Mass spectra of 4 μM KRas^G12C^ (KRas), 0.5 μM GFP, 4 μM sotorasib (S), and 2 μM Nb15 at equilibrium (top panel) and in a theta emitter (bottom panel). (F) Time-resolved native MS kinetic curves for (D) CA binding ethoxzolamide and KRas binding sotorasib. The concentrations listed account for a 2-fold dilution within the Taylor cone during rapid mixing. Deconvoluted and additional theta mass spectra for (A–C) are provided in Figures S22–24, respectively. Kinetic data is processed as described for Figure 3D, with determined kinetic parameters listed in the inset.

To further demonstrate the versatility of time-resolved native MS, we next examined the irreversible interaction between the oncogenic G12C mutant of KRas (KRas^G12C^) and its covalent inhibitor, sotorasib.^66^ Drug-bound KRas^G12C^, as well as Nb15-bound GFP, was detected in theta emitters and, as expected, the abundance of these complexes was lower than that observed under equilibrium conditions (Figure 4E). Irreversible inhibitors typically act through a two-step mechanism in which the drug first forms a rapid, reversible noncovalent complex with the protein, followed by a slower, rate-limiting covalent modification that renders the interaction irreversible. Given that native MS cannot distinguish the reversible and irreversible interactions, as both complexes have the same mass, and that the rate-limiting step is the irreversible interaction, we fit the data just for the irreversible interaction. Using the time-resolved native MS approach, we determined a *k*_on_ of 8 ± 2 × 10^5^ M^–1^ s^–1^ (Figure 4F), which is in close agreement with values determined in other buffer systems.^67^ Taken together, we demonstrate the ability to quantify the kinetics of noncovalent and irreversible small-molecule ligand binding to proteins.

## Discussion

We developed a time-resolved native MS approach that enables quantitative determination of biomolecular kinetics across a wide range of biomolecular interactions, such as for protein–protein and protein–drug interactions. To ensure accurate timing and quantification of biomolecular kinetics, we developed a mixing timer that enables precise determination of reaction times within theta emitters, allowing reliable measurement of kinetics under native conditions. This mixing timer is based on a nanobody–epitope interaction, which can, in principle, introduce differences in response factors between the apo and nanobody-bound states. However, no significant differences in the extracted kinetics were observed between response factor corrected and uncorrected data in this study. Future work should systematically evaluate response factor effects, particularly in systems involving larger mass differences, such as protein–protein interactions. In general, response factor corrections, for both the timer and the interaction of interest, should be considered when comparing species with substantially different masses or structures, as unequal signal responses and nonproportionality between intensity and concentration have been reported in native MS, especially for larger protein assemblies.^68–70^

We implemented this mixing timer by using two distinct strategies to enable time-resolved native MS measurements. The first is a fusion strategy in which the epitope is genetically fused to the protein of interest. The binding of the Nb to the fused epitope serves as a reporter for the mixing time. This approach is particularly advantageous for numerous workflows that rely on tags for affinity purification and those in which the analyte and nonfusion timer differ substantially in mass. However, this strategy likely requires recalibration of the epitope nanobody interaction for each fusion protein, as the fusion protein can affect the kinetics of the interaction, as observed here for the two different ALFA-tagged proteins. The second is a label-free, nonfusion strategy, in which the epitope and its corresponding Nb are introduced together with the protein of interest and its binding partner. This approach offers the advantage of not requiring a fused epitope tag, thereby eliminating the need to modify the expression constructs. It is particularly useful for proteins that are difficult to engineer or are isolated directly from native sources. For both strategies, the kinetics of the Nb–epitope interaction can be determined using conventional approaches, and these values provide a means to determine mixing times within θ-emitters.

Previous studies have demonstrated that mixing times in θ-emitters are influenced by different parameters and also the potential for accelerated reaction rates in microdroplets.^42,44,46,50,57^ However, if reactions are accelerated, then both the protein–ligand and timer interactions are expected to be equally accelerated, thereby preserving biologically relevant kinetics. Consistent with this expectation, the kinetic parameters determined by time-resolved native MS agree closely with those obtained here using BLI and with values reported previously.^52^ It is also not surprising to observe some differences between the two approaches. More specifically, BLI requires immobilization of one binding partner on the sensor surface, which can perturb apparent association and dissociation rates relative to solution-phase approaches in which both binding partners remain fully accessible, such as time-resolved native MS. Another key advantage of time-resolved native mass spectrometry is that it directly measures the mass of protein complexes rather than relying on optical signals, such as BLI, providing greater confidence in the results compared to traditional indirect methods. Beyond fundamental differences in the methods, difficulties arise in determining the kinetics of high-affinity systems. In this regime, small uncertainties in protein or ligand concentration can lead to disproportionately large differences in apparent kinetic parameters.^71–73^ Overall, the close agreement among time-resolved native MS, BLI, and prior reports indicates that reaction acceleration does not compromise the biological relevance of the kinetic parameters obtained here.

## Conclusions

Collectively, these studies establish a time-resolved native MS platform for quantifying the kinetics of biomolecular systems across diverse systems. Kinetic parameters determined by time-resolved native MS are consistent with established techniques, such as BLI, while requiring significantly less data acquisition time and eliminating the need for labeling or immobilization of either the protein or ligand. The approach also enables the determination of kinetics for high-affinity interactions that are otherwise difficult to characterize by using conventional native MS approaches. Moreover, its high mass precision enables the detection and quantification of binding partners spanning a broad range of sizes, ranging from small molecules to large protein assemblies. Beyond simple 1:1 protein–ligand systems, we envision that future work will extend this approach to capture complex, stepwise binding or assembly processes in real time, opening new opportunities to quantify binding kinetics in systems that have long been intractable using traditional methods. In closing, this time-resolved native MS approach transforms native MS from a static, equilibrium-focused technique to a dynamic platform capable of capturing the kinetics of biomolecular interactions.

## Experimental Section

### Protein Expression and Purification

All Nbs were expressed and purified as previously described.^74^ Briefly, codon-optimized genes for anti-alpha synuclein (NbAS),^75^ anti-GFP (Nb15),^52^ and anti-ALFA (NbALFA)^54^ were obtained from Twist Biosciences. For BLI experiments, a C-terminal AVI tag (GLNDIFEAQKIEWHE)^76^ was introduced using primers designed with the NEB BaseChanger Web site (NEB) and KLD enzyme mix (NEB). The Nb genes were cloned into a pET29b vector containing an N-terminal 10x His-tag and bdSUMO fusion. Shuffle T7 Express (NEB) cells were first transformed with the pRARE plasmid from Rosetta cells (Agilent) and then transformed with the Nb plasmids. A single colony was selected for kanamycin and chloramphenicol resistance, and grown in 50 mL of LB media overnight supplemented with 50 μg/mL of kanamycin and 35 μg/mL of chloramphenicol at 30 °C. 10 mL of the overnight culture was added to 1 L of LB. Cells were grown at 30 °C until an OD of 0.6–0.8 was reached, after which cells were induced with 0.5 mM IPTG and incubated at 18 °C overnight. Cells were harvested after 16 h and centrifuged at 4,000*g* for 10 min at 4 °C. Cells were resuspended in buffer A (20 mM Tris, pH 7.4, 150 mM NaCl, 20 mM imidazole, 10% glycerol) and lysed using four passages through a M-110P microfluidizer (Microfluidics) operating at 20,000 PSI. Insoluble material in the lysate was pelleted by centrifugation (20,000*g*, 4 °C, 20 min). The supernatant was filtered (0.45 μm syringe filter, Whatman) prior to loading onto a His-Trap HP 5 mL column (Cytiva) pre-equilibrated in loading buffer at room temperature. The Nb was eluted with 100% buffer B (buffer A with 500 mM imidazole) and desalted using a HiPrep 26/10 desalting column (GE Healthcare) pre-equilibrated with buffer A. The bdSUMO was cleaved by incubation with SENP1 (AddGene #104962, prepared following previously described protocols^77^) for 1 h at room temperature. The protein was then loaded on a 5 mL His-Trap, and the flow-through containing the Nb without the N-terminal fusion was collected.

The ALFA epitope was introduced to the C-terminus of superfolder GFP (sfGFP) and the N-terminus of SOS^cat^ (human SOS1, residues 558–1049, UniProt Q07889) using the NEB BaseChanger Web site (NEB) and KLD enzyme mix (NEB). sfGFP ALFA was cloned into a pCDF vector with a TEV cleavable N-terminal His-tag and transformed into Lemo21­(DE3) *Escherichia coli* cells. SOS^cat^ ALFA was cloned into a pET29 vector containing an N-terminal His-tag and transformed into Rosetta 2 DE3 cells (Novagen). For both sfGFP ALFA and SOS^cat^ ALFA, a single colony selected for streptomycin (sfGFP ALFA) or kanamycin/chloramphenicol (SOS^cat^ ALFA) resistance was grown in 50 mL of LB supplemented with 50 μg/mL of streptomycin or 50 μg/mL kanamycin/35 μg/mL chloramphenicol (SOS^cat^ ALFA) at 37 °C overnight. 7–10 mL of overnight culture was added to 1 L of LB supplemented with their respective antibiotic. Cells were incubated at 37 °C to an OD of 0.6–0.8 and induced with 0.5 mM IPTG. Cells were then incubated at 18 °C for 16 h. Cells were harvested by centrifugation (4,000*g* for 10 min at 4 °C) and resuspended in TBS (50 mM Tris, pH 7.4, 150 mM NaCl). Cells were then lysed as described above for the Nbs, and the lysate was centrifuged (20,000*g*, 4 °C, 20 min). sfGFP ALFA and SOS^cat^ ALFA were purified as described above for the Nbs, with the exception that the His-tag was not cleaved for SOS^cat^ ALFA.

The codon-optimized gene for CAII (thermostable)^78^ was synthesized (Twist Biosciences), and the gene for *E. coli* DHFR was a gift from Dr. Christian Hilty (Texas A&M University). These genes and for KRasG12C (residues 1–169)^79^ were cloned into a pET28 vector containing a C-terminal Strep-tag II and EPEA tag. All proteins were transformed into Lemo21­(DE3) *E. coli* cells, and expressed and lysed as described above, with the exception that kanamycin was used as the antibiotic. The clarified lysate was filtered prior to loading onto a StrepTrap HP 5 mL column (Cytiva) pre-equilibrated with TBS. The protein was eluted using TBS supplemented with 2.5 mM desthiobiotin. DHFR was fully loaded with NADPH (Cayman Chemicals) prior to MS analysis. For KRasG12C purification, all buffers were supplemented with 5 mM β-mercaptoethanol (β-ME), and the protein was loaded with GDP as previously described.^80^

eGFP (UniProt C5MKY7) modified with an N-terminal TEV protease cleavable His_8_ tag was cloned into a pACEbac1 (Geneva Biotech) insect cell expression plasmid, which was transformed into DH10EMBacY cells (Geneva Biotech), and blue/white screening was performed. A single white colony was inoculated overnight into 100 mL of LB, and baculoviral DNA was purified using a NucleoBond Xtra Midi kit (Macherey-Nagel). Thirty micrograms of DNA was mixed with 2 mL of phosphate-buffered saline (PBS) and 60 μL of 1 mg/mL PEI Max (Polysciences) transfection reagent. The mixture was then added to 30 mL of *Spodoptera frugiperda* (Sf9) cells in suspension and incubated at 27 °C. After 7 days, the virus was harvested by centrifugation at 4,000*g* for 10 min and the supernatant was recovered. A 50 mL culture of *Trichoplusia ni* cells was infected with 5 mL of the recombinant virus and grown at 27 °C for 72 h. The protein was harvested, lysed, and purified using a His-Trap as described above. The His-tag was then cleaved using TEV protease (1:100) at room temperature overnight. The protein solution was run over a HisTrap, and the flow-through containing tag-less protein was collected. All protein solutions were supplemented with 20% glycerol, flash frozen, and stored at −80 °C prior to experiments.

### Mass Spectrometry

All data was collected on an Exactive Plus Orbitrap Mass Spectrometer (Thermo Fisher Scientific, Bremen, German) with Extended Mass Range (EMR) mode on and no instrument modifications. Single (item #: BF150–110–10) and double (theta, item #: BT-150–10) barrel capillaries were obtained from Sutter Instruments. Both single and double (theta) barrel emitters were pulled using a Sutter P-1000 instrument with settings of heat 462 (ramp 447), pull 30, velocity 50, time 80, and pressure 500. All emitters were gold-coated and clipped to ∼20 μm (Figure S10) prior to MS analysis. Tips were imaged post-mortem by first drying overnight at 40 °C in a Thermo Scientific precision oven prior to imaging. Dried emitters were mounted at a 45° angle and immobilized with copper tape. Images were collected on a JEOL JCM-7000 Neo-Scope Benchtop SEM using an acceleration voltage of 15 kV, WD 13 mm, and a secondary electron detector. All proteins were buffer exchanged into 200 mM ammonium acetate using Micro Bio-Spin P6 Gel Columns (Bio-Rad Laboratories Inc., Hercules, CA). Methotrexate, chlorothiazide, and sotorasib were obtained from Cayman Chemicals, MedChem Express, and TargetMol, respectively. All ligands were diluted to 10 mM in DMSO for the stock solution. Protein concentrations were determined using a BCA assay (Pierce BCA Protein Assay Kit, Thermo Fischer Scientific). All proteins and ligands were diluted using 200 mM ammonium acetate to concentrations listed in figure captions prior to MS analysis. These concentrations were optimized to give kinetic information during the experiment, as well as to balance the signal from the timer and protein ligand system. Protein concentrations ranged from 400 nM to 4 μM, and under these conditions, significant nonspecific adduction is not anticipated.^68^ Moreover, the mass change associated with protein–ligand binding is relatively small (<5%), and therefore, no substantial deviation in their ionization efficiency is expected.^81^ The MS resolution was 8,750, and 10 μscans were used for all experiments. All other MS parameters are listed in Tables S2–S5. All MS parameters were optimized to provide the highest abundant complexes at equilibrium, thus ensuring that variation in the abundance of apo protein and protein complexes came only from differing mixing times.

### X-ray Structure of the Nb15–GFP Complex

Purified sfGFP and Nb15 were combined and loaded onto a HiLoad 16/600 Superdex 75 pg column (Cytiva) equilibrated in 20 mM Tris pH 7.4, 150 mM NaCl, and 10% glycerol. Fractions corresponding to the sfGFP–Nb15 complex were pooled and concentrated for crystallization trials. Initial crystallization screening was performed using a Mosquito LCP crystallization robot (TTP Labtech) in hanging-drop plates at 20 °C. Crystals were obtained from Index condition F8 (0.2 M ammonium sulfate, 0.1 M HEPES pH 7.5, and 25% (w/v) PEG 3350). The condition was further optimized by increasing the PEG 3350 concentration to 29%, which yielded larger crystals. Single crystals were mounted with CrystalCap HT Cryoloops (Hampton Research) and cryoprotected in a reservoir solution supplemented with 20% glycerol prior to flash freezing in liquid nitrogen. Diffraction data was collected in-house on a Rigaku R-axis-IV++. Phases were determined using molecular replacement with GFP (PDB code 8DHY) and an Nb15 model generated using the AlphaFold server.^82^ Model building and refinement were performed using Coot^83^ and Phenix.^84^

### Biolayer Interferometry

All proteins were diluted in 200 mM ammonium acetate to concentrations listed in Figures 3A,B and S12–S13 prior to BLI analysis. Prior to BLI experiments, the Nbs with AVI tags were biotinylated using previously established protocols, except BirA was expressed with a His_6_ tag instead of a GST tag.^85^ Samples were run over a HisTrap to capture the BirA and desalted to remove excess biotin prior to BLI analysis. Full biotinylation was confirmed with MS analysis. BLI experiments were performed on an 8-channel Octet R8 system (Sartorius) at 25 °C. After an initial baseline measurement, biotinylated proteins were loaded onto Octet Streptavidin (SA) Biosensors (Sartorius) at a wavelength of 1–2 nm. The sensors were then quenched with 10% bovine albumin serum in 200 mM ammonium acetate, followed by a second baseline measurement. Association with the binding partner was then performed for 300 s, followed by a 300 s dissociation step performed by moving the sensors to a well containing only buffer (200 mM ammonium acetate). A blank was used for baseline correction, and double referencing was performed to ensure nonspecific binding did not occur.

### Data Processing and Analysis

Data was first deconvoluted in the Unichrom module of Unidec,^86^ prior to kinetic analysis with custom Python scripts. Mole fraction data was first calculated for each species and converted to concentration. For a monomeric protein (P) binding one ligand (L), the differential equations are as follows:

1 $$\frac{\mathrm{dP}}{dt}=k_{\mathrm{off}}\left[\mathrm{PL}\right]-k_{\mathrm{on}}\left[P\right]\left[L\right]$$

2 $$\frac{\mathrm{dL}}{dt}=k_{\mathrm{off}}\left[\mathrm{PL}\right]-k_{\mathrm{on}}\left[P\right]\left[L\right]$$

3 $$\frac{d\mathrm{PL}}{dt}=k_{\mathrm{on}}\left[P\right]\left[L\right]-k_{\mathrm{off}}\left[\mathrm{PL}\right]$$

*K*_D_ can be determined using

4 $$K_{D}=\frac{k_{\mathrm{off}}}{k_{\mathrm{on}}}$$

An ordinary differential equations (ODE) solver was used to globally fit the kinetic model to the concentration time-dependent data determined from native MS. If the *K*_D_ is known, then the fitting parameters can be reduced by substituting one of the variables, such as *k*_off_ with *k*_on_·*K*_D_. To account for dilution after rapid mixing, we introduced a mixing factor (α) to adjust the initial concentration of P and L

5 $${\left[P\right]}_{o}={\propto \left[P\right]}_{\mathrm{total}}$$

6 $${\left[L\right]}_{o}={\left(1-\propto \right)\left[L\right]}_{\mathrm{total}}$$

For example, an α factor of 0.5 represents a 2-fold dilution or 1:1 mixing. To ensure that the use of an α factor per burst as compared to per emitter was statistically justified, we implemented the classical nested-model *F*-test for the Nb15 binding GFP data (Figure 3) in Python as previously described.^87,88^ The *F-*test revealed that the inclusion of additional fitting parameters was statistically justified.

All curves were manually inspected for accurate fitting, and those deviating significantly from the fit were discarded (Figure S25). All custom Python code is available on GitHub (https://github.com/LaganowskyLab). BLI data was processed using the Octet analysis software (Sartorius) with global fitting, a 1:1 interaction model, and baseline correction from the blank as previously described.^89^ The reported kinetic parameters (*k*_off_, *k*_on_, and *K*_D_) were determined from five independent emitter replicates, each containing at least three bursts. Values of *k*_off_ and *k*_on_ were obtained from fitting, and *K*_D_ was calculated for each replicate from *k*_off_ and *k*_on_ as described above. Reported uncertainties represent the mean ± standard deviation across replicates.

## Supplementary Material



## Data Availability

The GFP–Nb15 structure has been deposited with accession code 9ZG5. All other study data are included in the article and/or Supporting Information.

## Notes

Comparison of a theta and standard emitter, schematic of fusion timer method, mass spectra showing the influence of backing pressure and tip diameter on mixing times, mixing times for a burst of mass spectra as determined by the GFP–NbAS interaction, comparison of GFP–Nb15 abundance for varying emitter diameter, deconvoluted and raw equilibrium and theta mass spectra for systems timed with GFP–NbALFA and SOS^cat^–NbALFA, response factor calibration curve for GFP–NbALFA, response factor corrected kinetics for GFP–NbALFA, deconvoluted spectra for all raw spectra shown in the main text, additional raw and deconvoluted mass spectra for all three protein–drug interactions, examples of discarded kinetic data, X-ray crystallography parameters for the GFP–Nb15 structure, and MS parameters for all experiments (PDF)
