# Supplementary material for: Time-Resolved Native Mass Spectrometry for Direct Measurement of Biomolecular Kinetics
Source: J Am Chem Soc. 2026 May 9;148(19):19567–77. doi: 10.1021/jacs.5c21842 (PMC13195665; doi:10.1021/jacs.5c21842)
Supplement: Supplementary file 1 [file ja5c21842_si_001.pdf]

Supporting Information

Time-resolved native mass spectrometry for direct measurement of biomolecular kinetics

Virginia K. James,[1] Lauren Stover,[1] Hanieh Bahramimoghaddam,[1] Tanishq Khandelwal,[1] Jing-Yuan Chang,[1] James Downing,[1] Elena Scott,[1] Kathleen O. Bailey,[1] David H. Russell,[1] Lane A. Baker,[1] Arthur Laganowsky,[1]*

[1] Department of Chemistry, Texas A&M University, College Station, TX, 77843, USA

*Correspondence to alaganowsky@chem.tamu.edu

**Figure S1.** Mixing in a theta emitter as compared to a standard emitter.	S3
**Figure S2.** An overview of time-resolved native mass spectrometry approach and determination of biomolecular kinetics using an internal timer.	S4
**Figure S3.** Theoretical protein-ligand binding curves for a protein-ligand interaction with the same K_D and different k_{on}	S5
**Figure S4.** Mass spectra of eGFP binding NbAS (AS) and sfGFP binding NbALFA at equilibrium.	S6
**Figure S5.** Mass spectra of SOS^{cat} binding NbAS (AS) and GFP binding Nb15 at equilibrium.	S7
**Figure S6.** Influence of experimental parameters on mixing time.	S8
**Figure S7.** Abundance of sfGFP ALFA, NbALFA, eGFP, and NbAS over three ESI power cycles. Raw and deconvoluted spectra of sfGFP ALFA binding NbALFA and eGFP binding NbAS in a theta emitter.	S9
**Figure S8.** Abundance of SOS^{cat} ALFA, NbALFA, eGFP, and Nb15 over three ESI power cycles. Raw and deconvoluted spectra of SOS^{cat} ALFA binding NbALFA and eGFP binding Nb15 in a theta emitter.	S10
**Figure S9.** Deconvoluted mass spectra of GFP binding Nb15 and NbAS at equilibrium and in a theta emitter.	S11
**Figure S10.** SEM images of two clipped theta emitters.	S12
**Figure S11.** Mole fractions of mixtures of GFP and Nb15 collected at equilibrium with varying emitter diameters.	S13
**Figure S12.** Biolayer interferometry (BLI) sensorgrams showing SOS^{cat} ALFA binding to biotinylated NbALFA.	S14
**Figure S13.** Biolayer interferometry (BLI) sensorgrams showing sfGFP ALFA binding to biotinylated NbALFA.	S15
**Figure S14.** Crystal structure of the GFP-Nb15 complex.	S16
**Figure S15.** Concentration of the GFP-NbAS complex (dots) plotted as a function of calibrated mixing time.	S17
**Figure S16.** Time resolved native MS kinetic curve for the Nb15-GFP interaction fit with no, one, and burst variable alpha factors.	S18

**Figure S17.** Theoretical abundance curves for NbAS bound GFP with different mixing (α) factors.	S19
**Figure S18.** Time-resolved native MS kinetic curves showing GFP binding to Nb15, timed using SOS^{cat} ALFA binding NbALFA.	S20
**Figure S19.** Time-resolved native MS kinetic curves showing eGFP binding to NbAS, timed using sfGFP ALFA binding NbALFA.	S21
**Figure S20.** Mole fractions of mixtures of the purified complex GFP ALFA-NbALFA and apo GFP ALFA at varying concentrations of apo GFP ALFA determined using solution UV assay vs native MS.	S22
**Figure S21.** Kinetics for uncorrected and response factor corrected GFP binding NbAS.	S23
**Figure S22.** Deconvoluted mass spectra of CA binding E and GFP binding Nb15 at equilibrium. Abundance of each species over three ESI power cycles. Raw and deconvoluted spectra of CA binding E and GFP binding Nb15 in a theta emitter.	S24
**Figure S23.** Deconvoluted mass spectra of DHFR binding M and GFP binding Nb15 at equilibrium. Abundance of each species over three ESI power cycles. Raw and deconvoluted spectra of DHFR binding M and GFP binding Nb15 in a theta emitter.	S25
**Figure S24.** Deconvoluted mass spectra of KRas binding S and GFP binding Nb15 at equilibrium. Abundance of each species over three ESI power cycles. Raw and deconvoluted spectra of KRas binding S and GFP binding Nb15 in a theta emitter.	S26
**Figure S25.** Examples of discarded curves	S27
**Table S1.** Summary of X-ray data collection and refinement statistics.	S28
**Table S2.** Kinetic parameters for all Nb systems.	S29
**Table S3.** MS parameters for GFP-EPEA binding to NbAS and Nb15 data collection as shown in Figure 2.	S30
**Table S4.** MS parameters for CA data collection as shown in Figure 3A.	S31
**Table S5.** MS parameters for DHFR data collection as shown in Figure 3B.	S32
**Table S6.** MS parameters for KRasG12C data collection as shown in Figure 3C.	S33

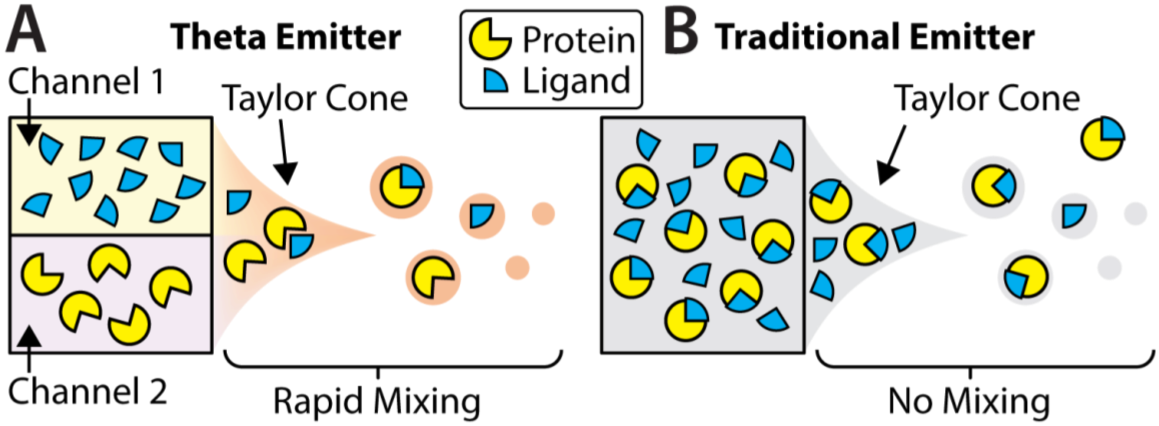

Figure S1. (A) Mixing in a theta emitter as compared to (B) a standard emitter.

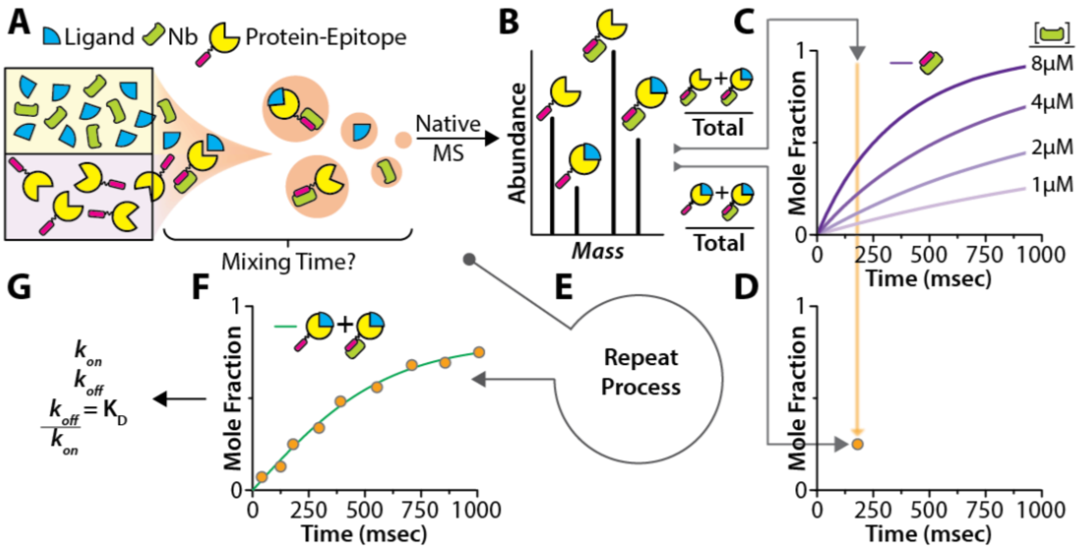

Figure S2. An overview of time-resolved native mass spectrometry approach and determination of biomolecular kinetics using the fusion approach. A) Schematic of the theta emitter configuration illustrating the dual-channel electrospray setup, where one channel contains the protein fused to an epitope tag and the second channel contains the corresponding Nb and ligand. Rapid mixing occurs at the emitter tip prior to ionization and transfer into the mass spectrometer. (B-G) Workflow of the time-resolved native MS approach, where the Nb-epitope interaction serves as an internal timer to calibrate mixing times for each mass spectrum. C) Theoretical time-dependent binding curves for 1 µM protein containing an epitope binding to different initial concentrations of Nb based on a k_{on} of 3.6 x 105 ($M^{-1}s^{-1}$) and k_{off} of 9.4 x 10-6 (s^{-1}).

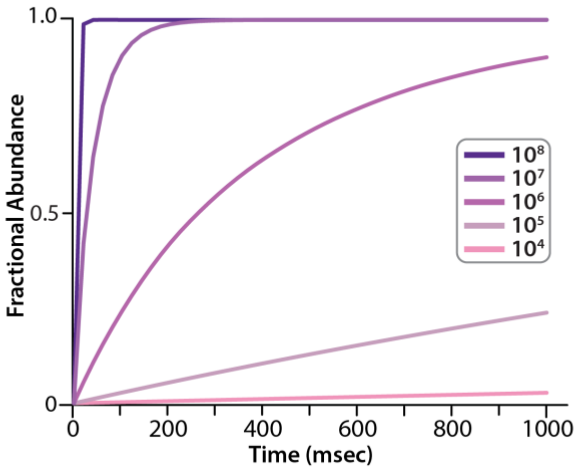

Figure S3. Theoretical protein-ligand binding curves for a protein-ligand interaction with a K_D of 500 nM with different k_{on} ($M^{-1}s^{-1}$) values (shown in legend).

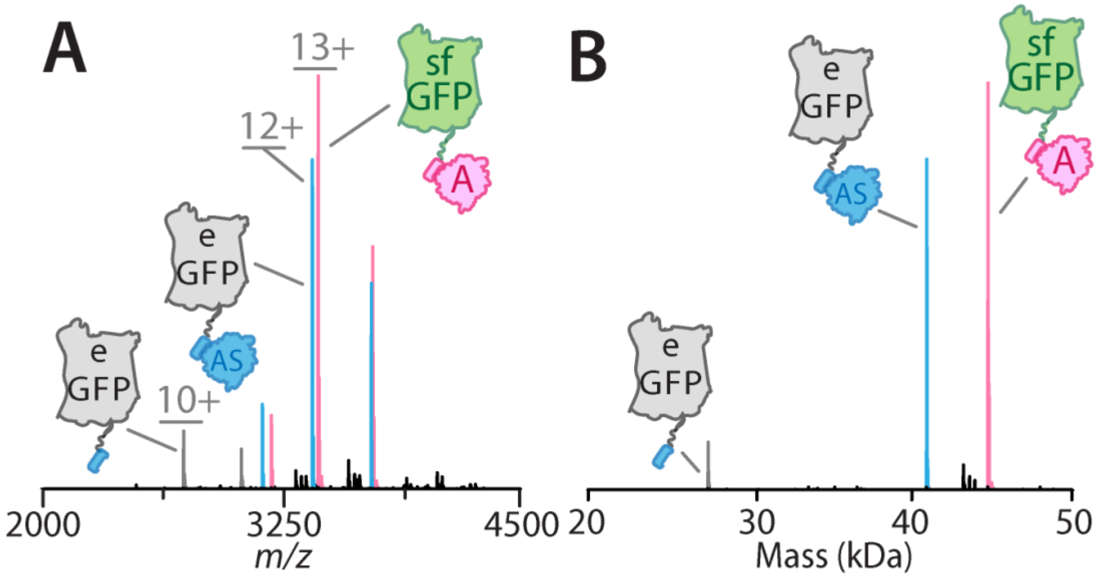

Figure S4. (A) Raw and (B) deconvoluted mass spectra of 0.6 µM eGFP binding 1 µM NbAS (AS) and 0.8 µM sfGFP binding 3 µM NbALFA (A) at equilibrium.

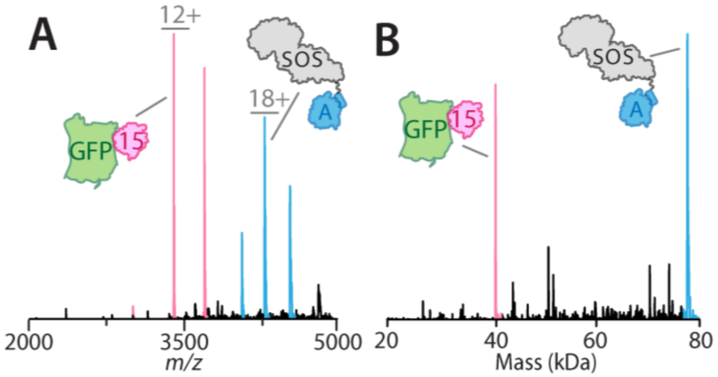

Figure S5. (A) Raw and (B) deconvoluted mass spectra of 0.6 µM eGFP binding 1.33 µM Nb15 (15) and 2 µM SOS^{cat} ALFA binding 2 µM NbALFA (A) at equilibrium.

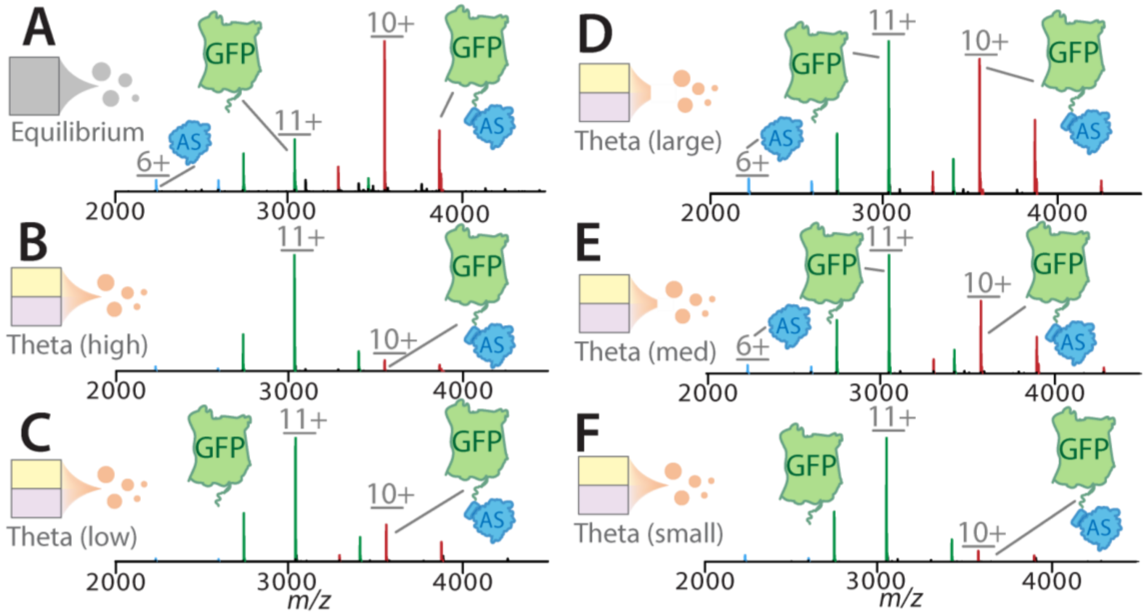

Figure S6. Influence of experimental parameters on mixing time. A) 0.8 µM green fluorescent protein (GFP) binding and 2 µM NbAS (AS) at equilibrium. B-F) GFP binding NbAS in a theta emitter under (B) high and (C) low backing pressure, and with a (D) large, (E) medium, and (F) small tip diameter.

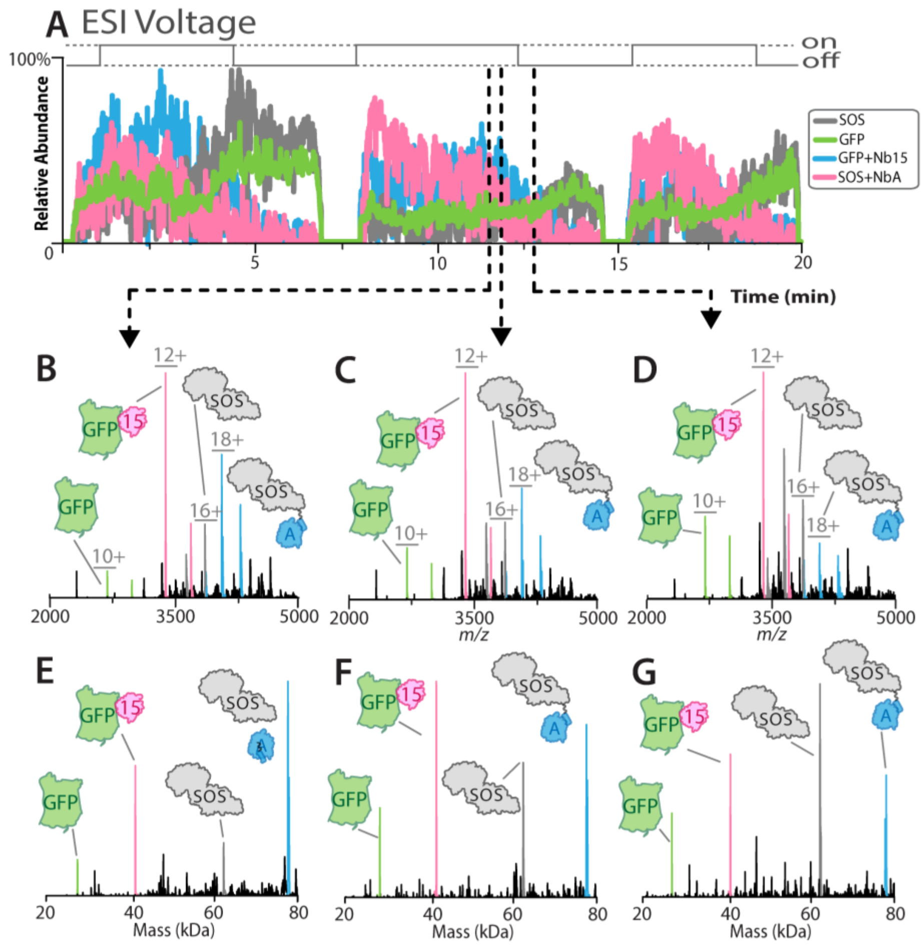

Figure S7. A) Extracted mass chromatograms of SOS^{cat} ALFA, SOS^{cat} ALFA bound to NbALFA, eGFP and eGFP bound Nb15 over three ESI power cycles. C-E) 0.6 µM eGFP binding 1 µM NbAS (AS) and 0.8 µM sfGFP binding 3 µM NbALFA (A) in a theta emitter (C) 3 minutes after ESI voltage was turned on, (D) after 3.75 minutes of sustained voltage, and (E) after 4 minutes of sustained voltage. Concentrations listed account for a twofold dilution within the Taylor cone during rapid mixing. Deconvoluted mass spectra for C, D, and E are shown in F, G, and H, respectively.

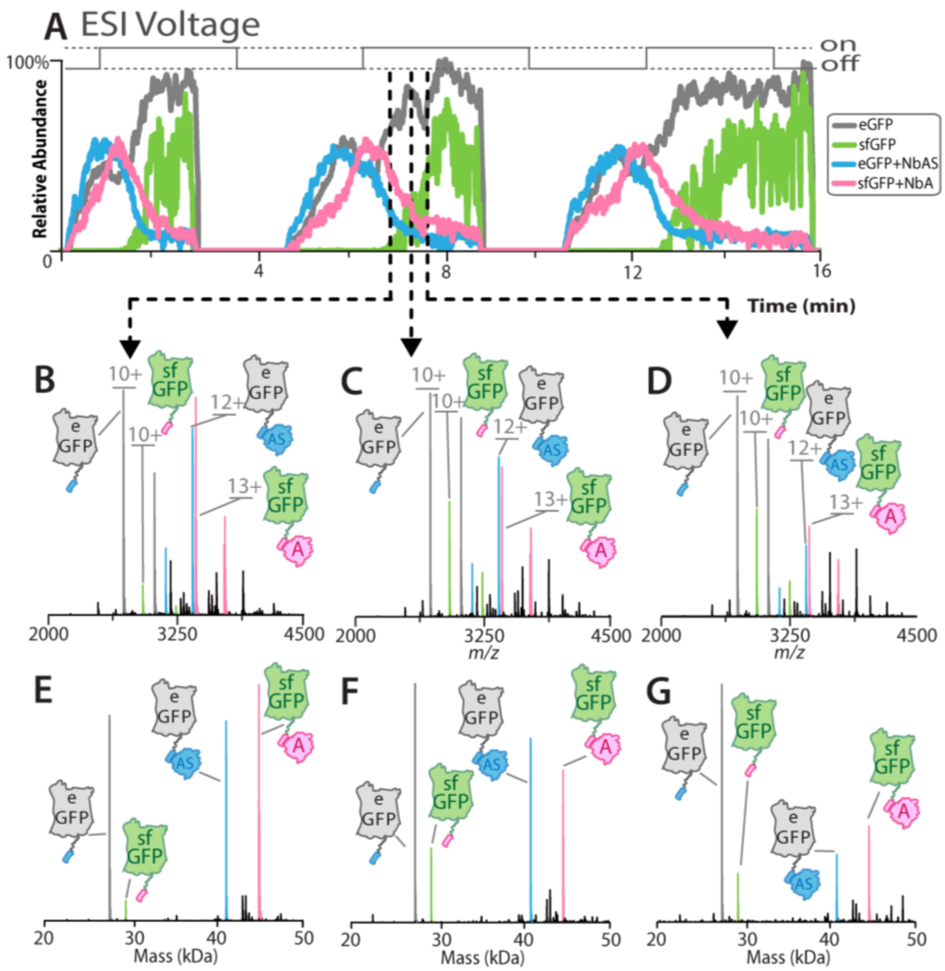

Figure S8. A) Extracted mass chromatograms of sfGFP ALFA, sfGFP ALFA bound to NbALFA, eGFP and eGFP bound NbAS over three ESI power cycles. C-E) 2 µM sfGFP ALFA binding 2 µM NbALFA and 0.4 µM eGFP binding 2 µM NbAS in a theta emitter (C) 3 minutes after ESI voltage was turned on, (D) after 3.75 minutes of sustained voltage, and (E)after 4 minutes of sustained voltage. Concentrations listed account for a twofold dilution within the Taylor cone during rapid mixing. Deconvoluted mass spectra for C, D, and E are shown in F, G, and H, respectively.

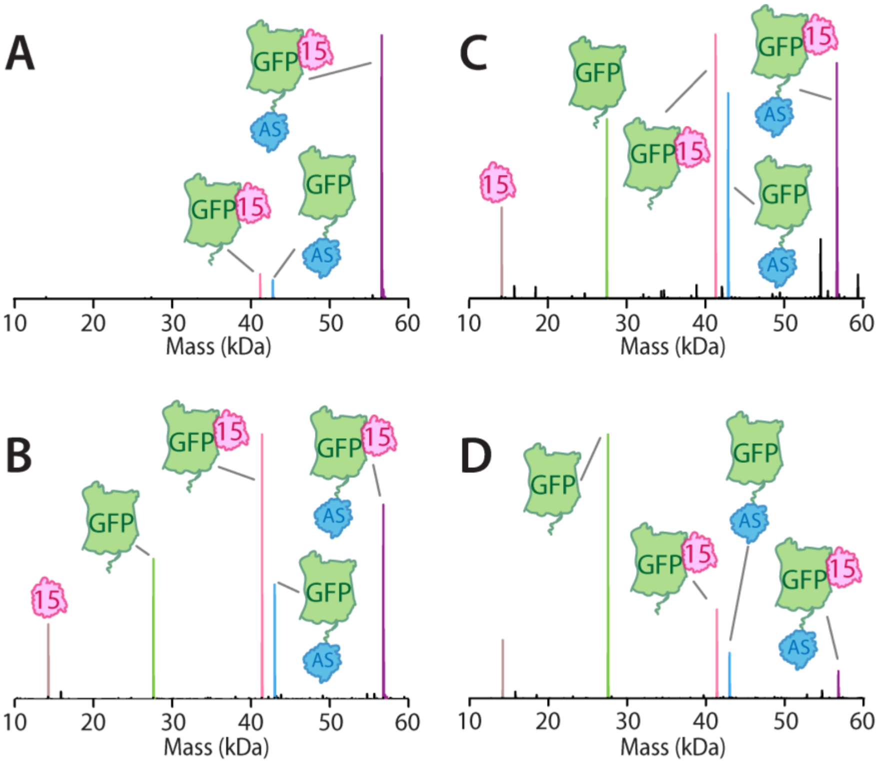

Figure S9. A) Deconvoluted mass spectra of 0.8 µM green fluorescent protein (GFP) binding 2 µM Nb15 (15) and 2 µM NbAS (AS) at equilibrium (raw spectra shown in Figure 2A). B-D) Deconvoluted mass spectra 0.8 µM GFP binding 2 µM Nb15 and 2 µM NbAS in a theta emitter shortly (C) after ESI voltage was turned on, (D) after a minute of sustained voltage, and (E) after 1.5 minutes of sustained voltage.

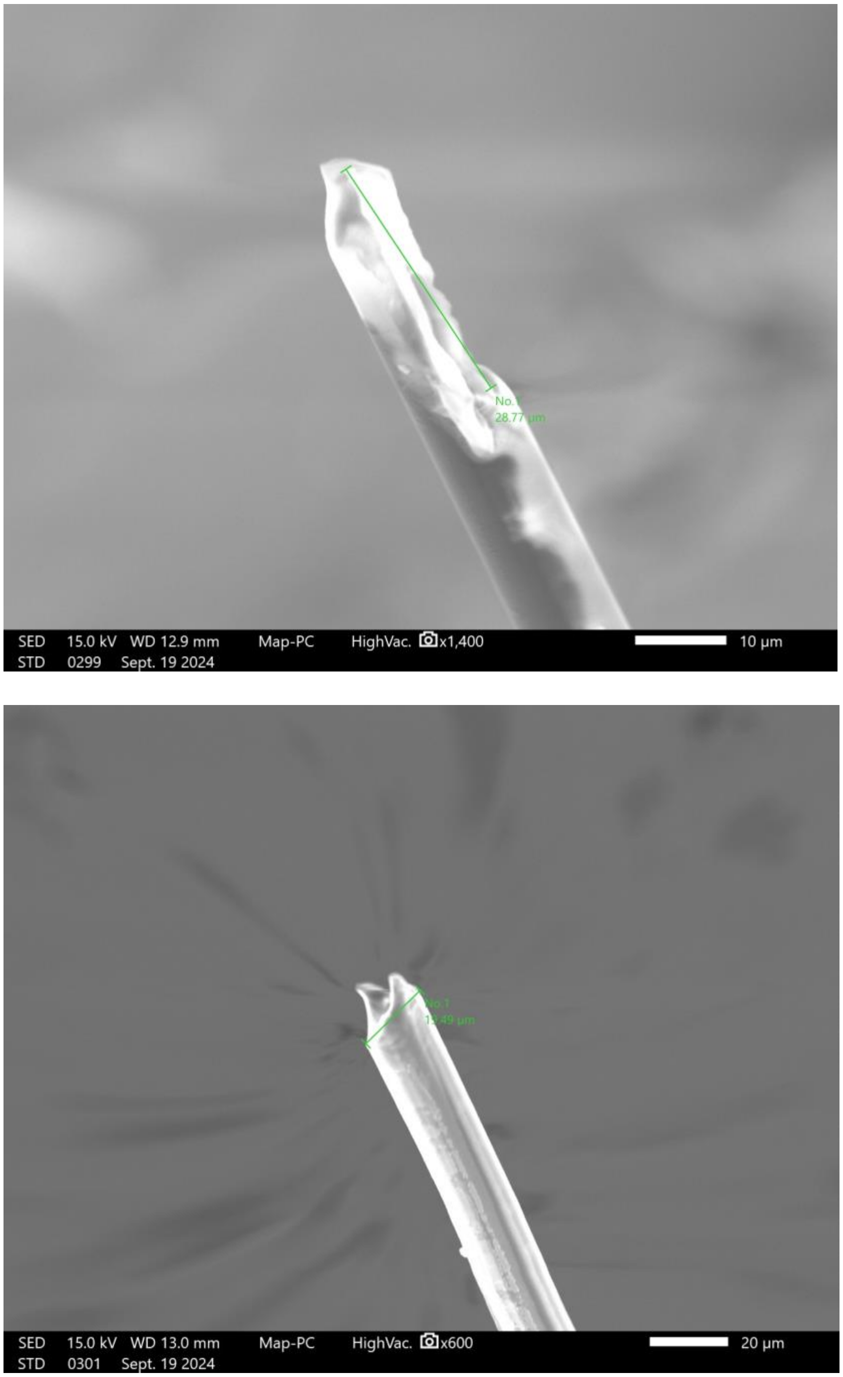

Figure S10. SEM images of two clipped theta emitters.

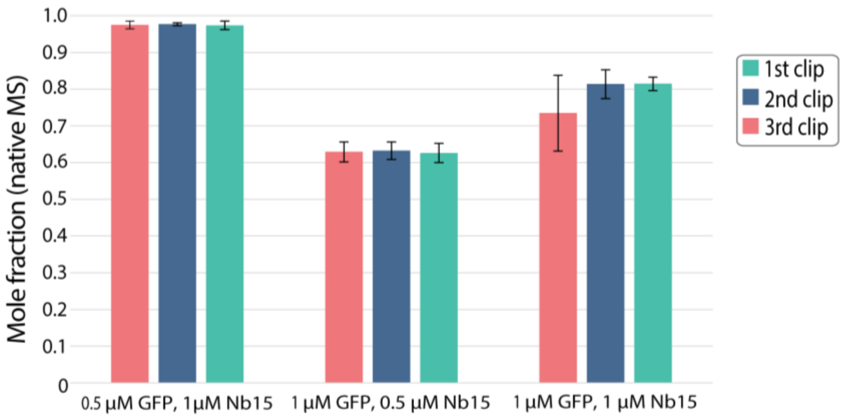

Figure S11. Mole fractions of (A) 0.5 µM GFP and 1 µM Nb15, (B) 1 µM GFP and 0.5 µM Nb15, and (C) 1 µM GFP and 1 µM Nb15 collected at equilibrium with varying emitter diameters. Each clip break represents approximately 1 mm removed from the end of the tip.

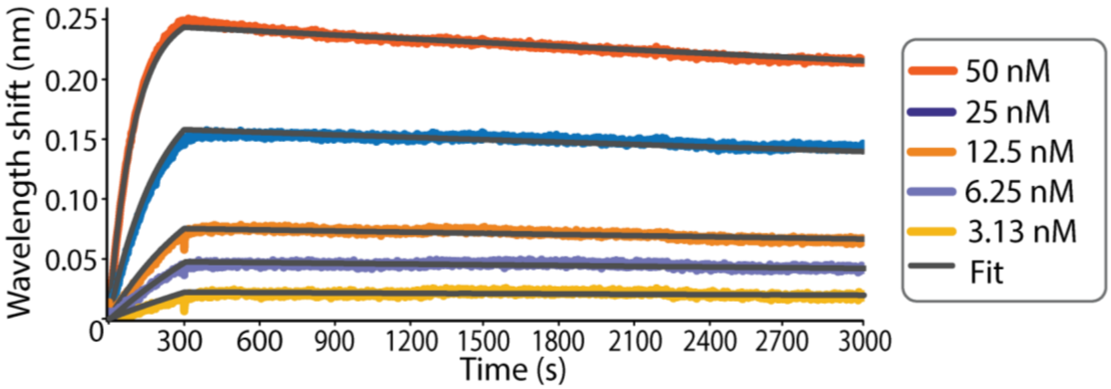

Figure S12. Biolayer interferometry (BLI) sensorgrams showing SOS^{cat} ALFA binding to biotinylated NbALFA with corresponding global fits (solid lines) based on a 1:1 binding model yielding a k_{on} of 1.931±0.002 × 10^5 $M^{-1}s^{-1}$, k_{off} of 4.531±0.008 × 10^{-5} s^{-1}, and K_D of 234.7±0.5 pM.

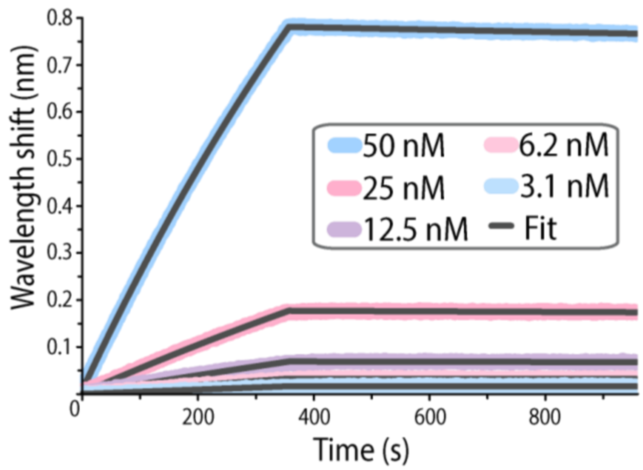

Figure S13. Biolayer interferometry (BLI) sensorgrams showing GFP ALFA binding to biotinylated NbALFA with corresponding global fits (solid lines) based on a 1:1 binding model yielding a k_{on} of 2.256±0.003 × 10^4 $M^{-1}s^{-1}$, k_{off} of 3.11±0.02 × 10^{-5} s^{-1}, and K_D of 1.379±0.007 nM.

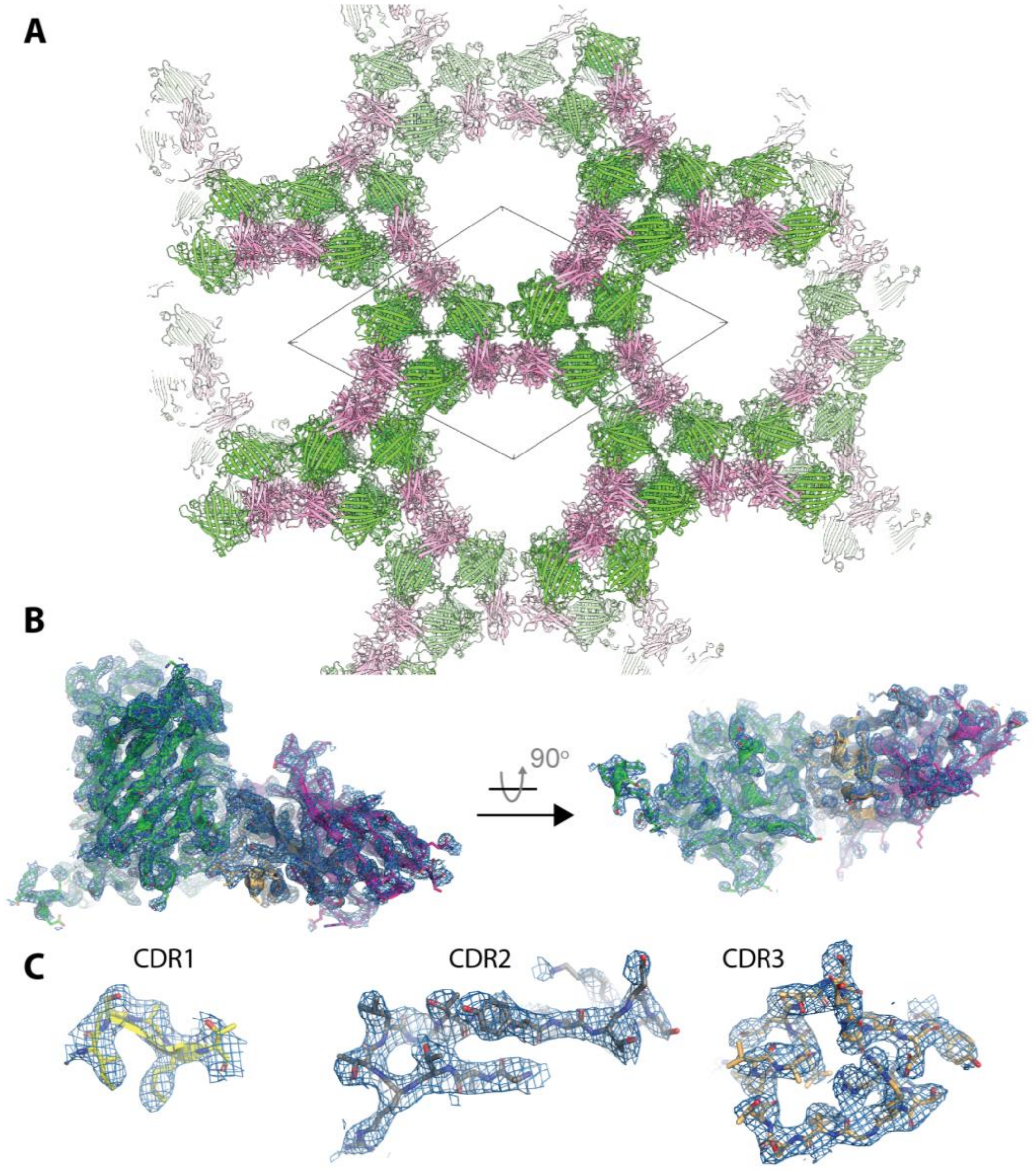

Figure S14. Crystal structure of the GFP-Nb15 complex. A) Crystal packing with structure shown in cartoon representation. GFP and Nb15 are shown in green and pink, respectively. B) Structure of the complex shown in stick representation with 2Fo–Fc electron density map contoured at 1 sigma. C) Views of the complementary determining regions of Nb15 shown as described in panel A. Figure prepared using ChimeraX and Pymol.

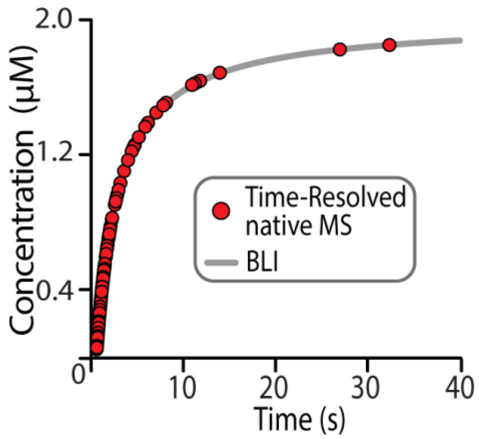

Figure S15. Concentration of the GFP-NbAS complex (dots) plotted as a function of calibrated mixing time, determined from BLI kinetics (**Figure 3D**) for the GFP-NbAS interaction. Selected mass spectra along this curve are shown in **Figure 3C-E** (raw) and **Figure S9B-D** (deconvoluted).

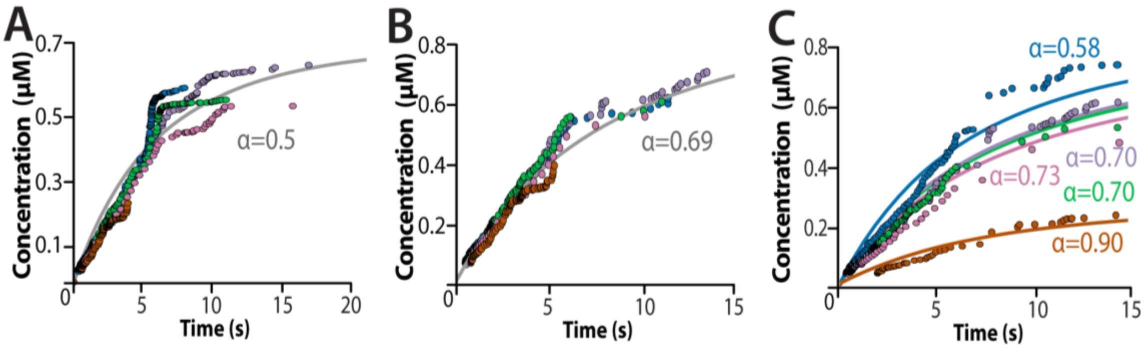

Figure S16. Time resolved native MS kinetic curve for the GFP-Nb15 interaction fit (A) without any alpha factor, *i.e.* assuming perfect mixing, (B) with one alpha factor for all bursts, and (C) one alpha factor per burst as seen in **Figure 3D**.

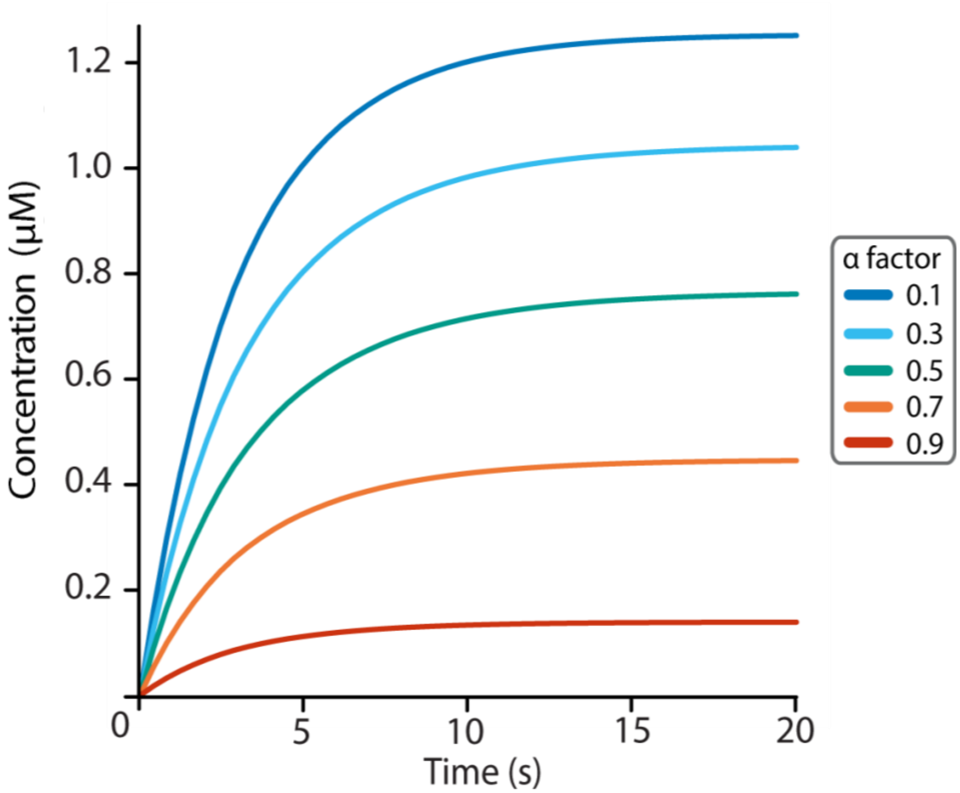

Figure S17. Theoretical abundance curves for NbAS bound GFP with different mixing (α) factors.

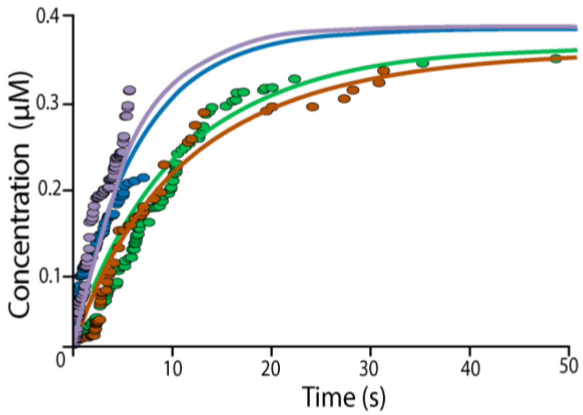

Figure S18. Time-resolved native MS kinetic curves showing GFP binding to Nb15, timed using SOS^{cat} ALFA binding NbALFA. Data from five bursts are plotted in distinct colors, fit to a 1:1 global kinetic model in which each burst is assigned its own mixing factor (α). The colored traces represent individual bursts, and solid lines denote the corresponding model fits, with each α value labeled in the matching color.

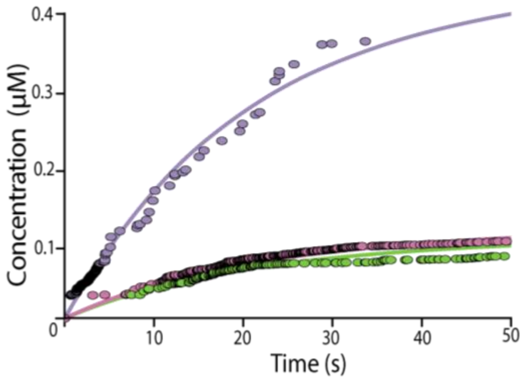

Figure S19. Time-resolved native MS kinetic curves showing eGFP binding to NbAS, timed using sfGFP ALFA binding NbALFA. Data from five bursts are plotted in distinct colors, fit to a 1:1 global kinetic model in which each burst is assigned its own mixing factor (α). The colored traces represent individual bursts, and solid lines denote the corresponding model fits, with each α value labeled in the matching color.

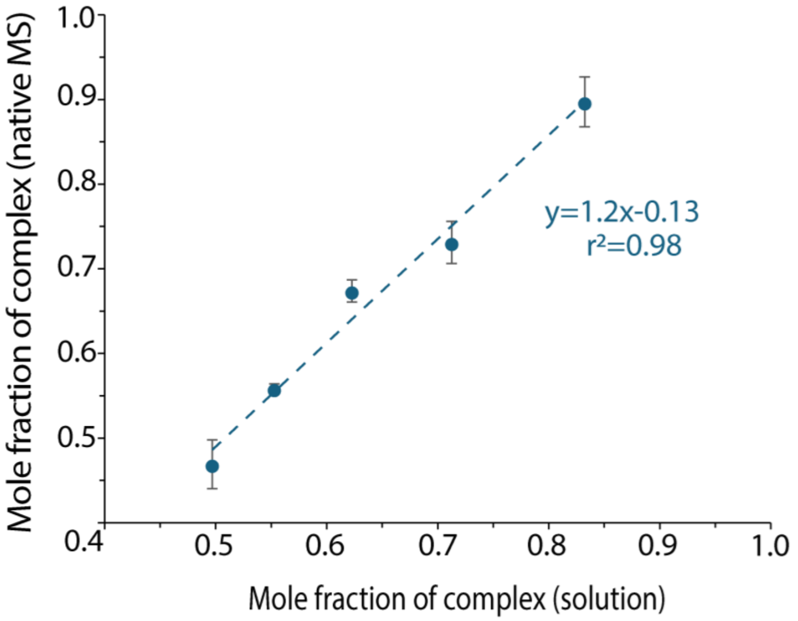

Figure S20. Mole fractions of mixtures of the purified complex GFP ALFA-NbALFA and apo GFP ALFA at varying concentrations of apo GFP ALFA determined using solution UV assay vs native MS. The GFP ALFA-NbALFA was purified by loading his tag GFP ALFA incubated with excess NbALFA onto a his trap. Full binding of GFP ALFA to NbALFA was confirmed with native MS prior to adding apo GFP ALFA.

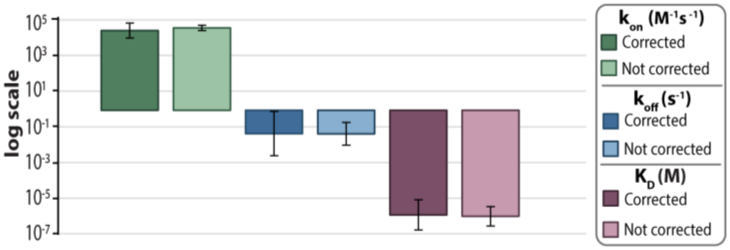

Figure S21. Kinetic parameters and equilibrium dissociation constant (K_D) for the GFP-Nb15 interaction determined using time-resolved native MS when accounting for response factor for the timer complex **Figure S20** (corrected) and not accounting for response factor (uncorrected).

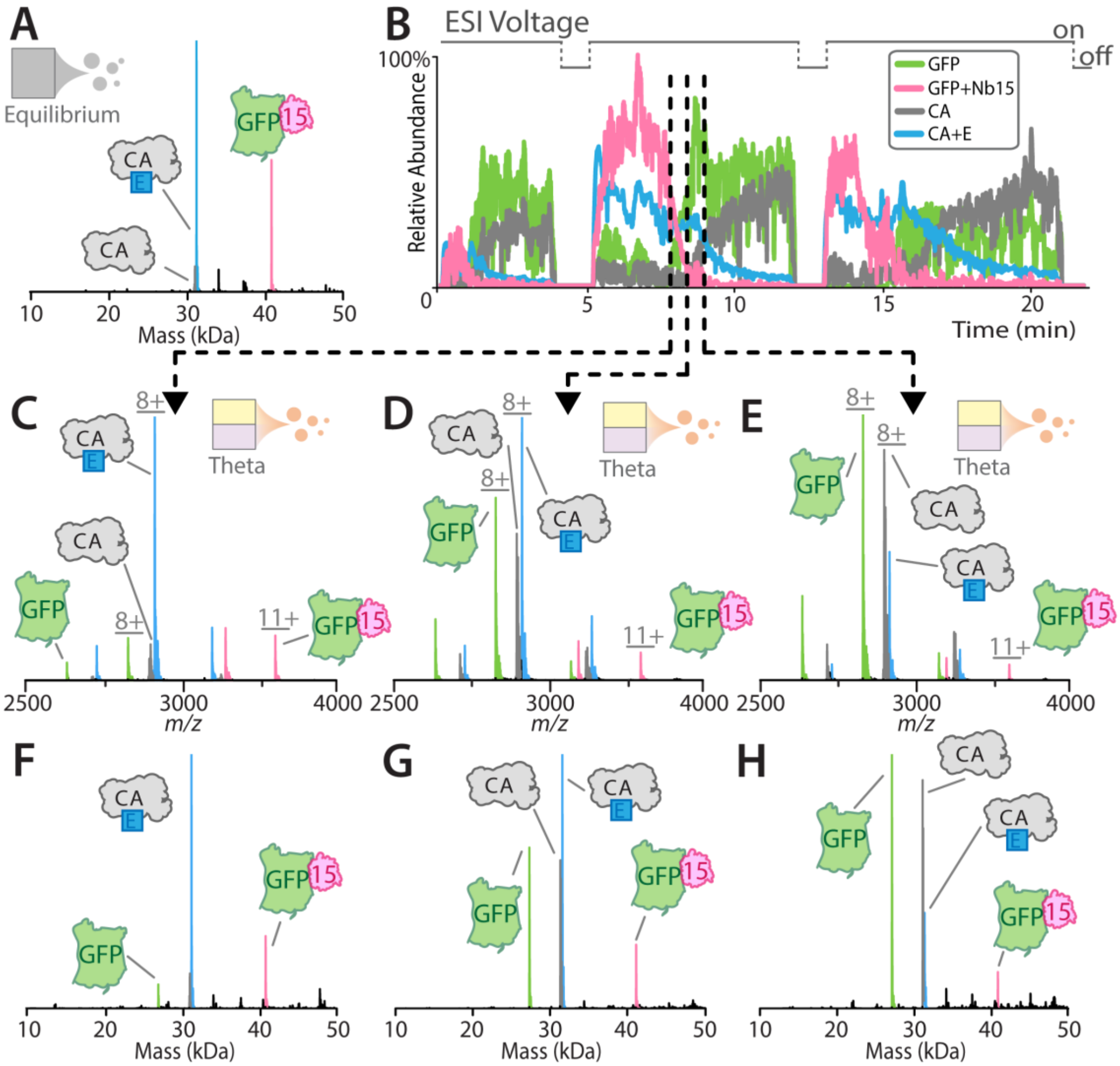

Figure S22. A) Deconvoluted mass spectra of 2 µM carbonic anhydrase (CA) binding 2 µM ethoxzolamide (E) and 0.4 µM green fluorescent protein (GFP) binding 2 µM Nb15 (15) at equilibrium (raw spectra shown in Figure 3A). B) Extracted mass chromatograms of CA, CA bound to E, GFP and GFP bound Nb15 over three ESI power cycles. C-E) 2 µM CA binding 2 µM ethoxzolamide and 0.4 µM GFP binding 2 µM Nb15 in a theta emitter (C) shortly after ESI voltage was turned on, (D) after 3 minutes of sustained voltage, and (E) after 3.5 minutes of sustained voltage. Concentrations listed account for a twofold dilution within the Taylor cone during rapid mixing. Deconvoluted mass spectra for C, D, and E are shown in F, G, and H, respectively.

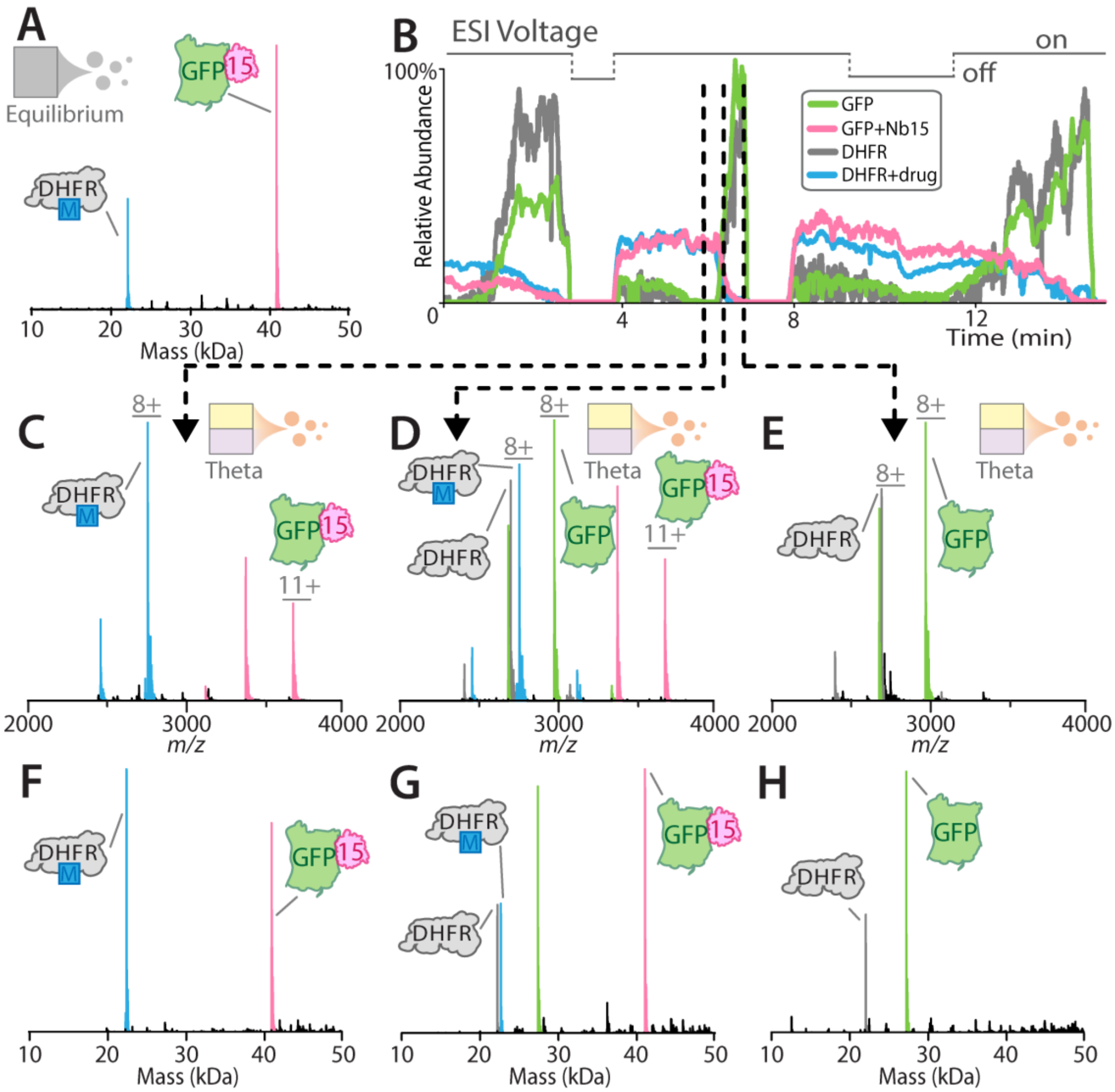

Figure S23. A) Deconvoluted mass spectra of 1 µM dihydrofolate reductase (DHFR) binding 1 µM methotrexate (M) and and 0.5 µM green fluorescent protein (GFP) binding 1 µM Nb15 (15) at equilibrium (raw spectra shown in Figure 3B). B) Extracted mass chromatograms of DHFR, DHFR bound to M, GFP and GFP bound Nb15 over three ESI power cycles. C-E) 1 µM DHFR binding 1 µM methotrexate and 0.5 µM GFP binding 1 µM Nb15 at equilibrium in a theta emitter (C) shortly after ESI voltage was turned on, (D) after 2 minutes of sustained voltage, and (E) after 2.5 minutes of sustained voltage. Concentrations listed account for a twofold dilution within the Taylor cone during rapid mixing. Deconvoluted mass spectra for C, D, and E are shown in F, G, and H, respectively.

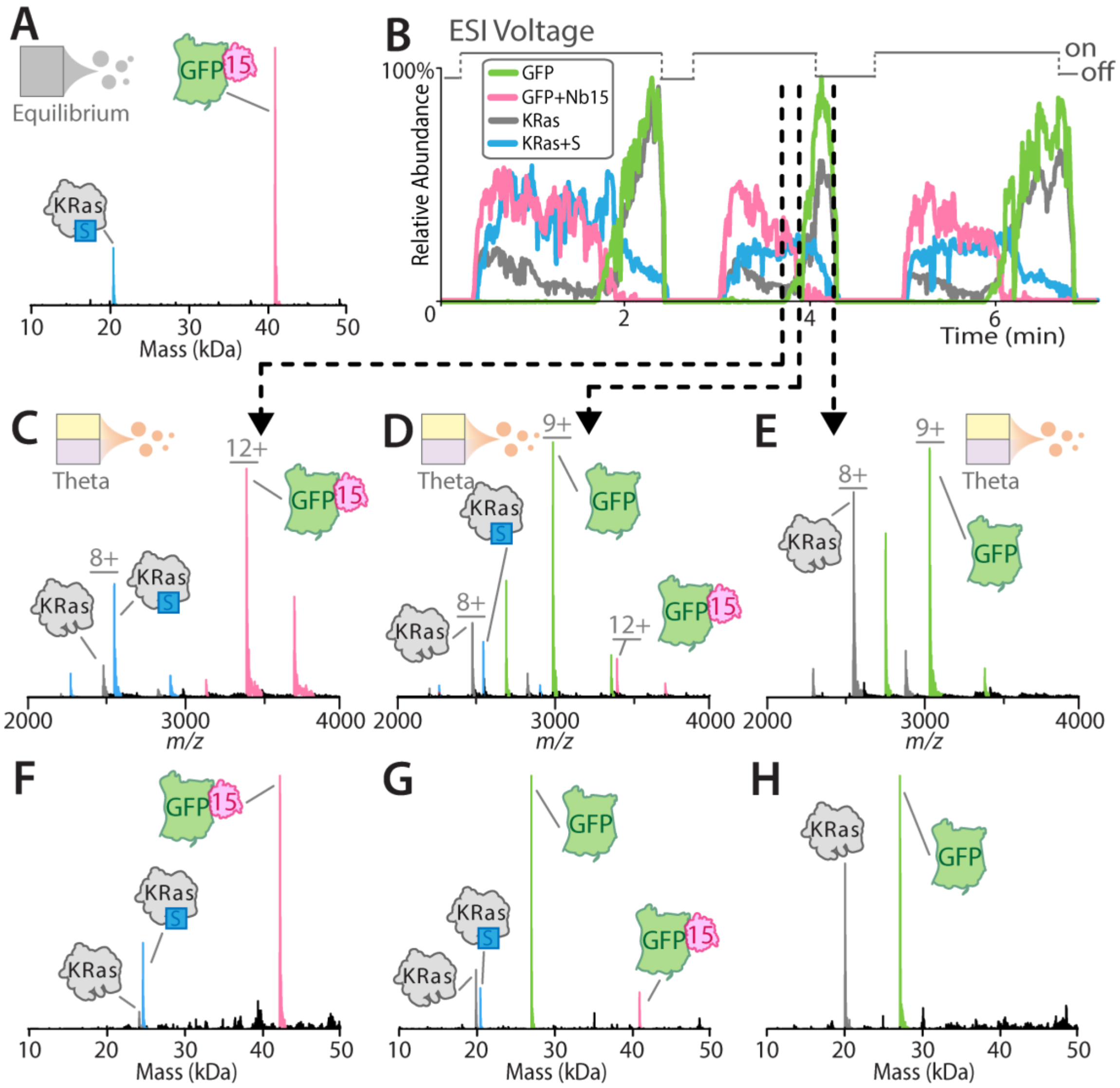

Figure S24. A) Deconvoluted mass spectra of 4 µM KRas^{G12C} (KRas) binding 4 µM sotorasib (S) and 0.5 µM µM green fluorescent protein (GFP) binding 2 µM Nb15 (15) at equilibrium (raw spectra shown in Figure 3C). B) Extracted mass chromatograms of KRas, KRas bound to sotorasib, GFP and Nb15 bound GFP over three ESI power cycles. C-E) 4 µM KRas binding 4 µM sotorasib and 0.5 µM GFP binding 2 µM Nb15 in a theta emitter (C) shortly after ESI voltage was turned on, (D) after 2 minutes of sustained voltage, and (E) after 2.5 minutes of sustained voltage. Concentrations listed account for a twofold dilution within the Taylor cone during rapid mixing. Deconvoluted mass spectra for C, D, and E are shown in F, G, and H, respectively.

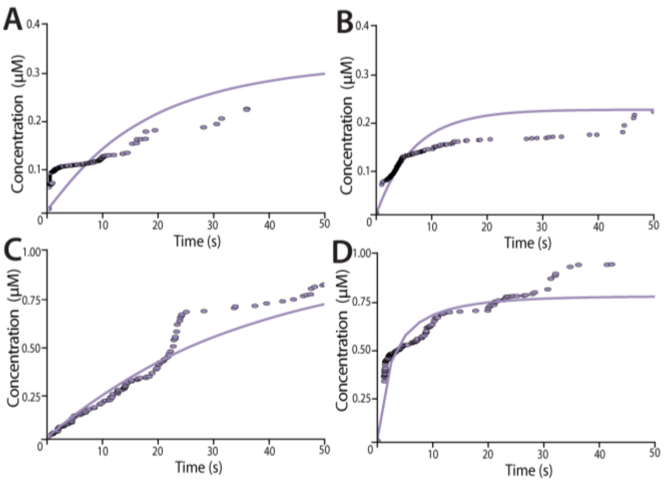

Figure S25. Examples of discarded curves for (A-B) GFP EPEA binding NbAS and (C-D) DHFR binding methotrexate.

Table S1. Summary of X-ray data collection and refinement statistics.

	GFP-Nb15 complex
**Wavelength (Å)**	1.5418
**Resolution range (Å)**	34.4 - 3.4 (3.51 - 3.4)
**Space group**	P 32
**Unit cell**	169.05 169.05 70.79 90 90 120
**Total reflections**	172412 (14781)
**Unique reflections**	88469 (7658)
**Multiplicity**	1.9 (1.9)
**Completeness (%)**	99.80 (99.89)
**Mean I/sigma(I)**	2.37 (0.94)
**Wilson B-factor**	27.60
**R-merge**	0.3438 (0.8434)
**CC1/2**	0.835 (0.27)
**CC***	0.954 (0.652)
**Reflections used in refinement**	31028 (2845)
**Reflections used for R-free**	1552 (142)
**R-work**	0.2949 (0.3089)
**R-free**	0.3313 (0.3613)
**Number of non-hydrogen atoms**	11330
**macromolecules**	11227
**ligands**	103
**Protein residues**	1447
**RMS(bonds)**	0.002
**RMS(angles)**	0.46
**Ramachandran favored (%)**	96.28
**Ramachandran allowed (%)**	3.51
**Ramachandran outliers (%)**	0.21
**Rotamer outliers (%)**	0.00
**Clashscore**	5.02
**Average B-factor**	22.54
**macromolecules**	22.56
**ligands**	20.79
**PDB Code**	9ZG5

Table S2. Comparison of kinetic values for all Nbs.

		Nb15 (GFP-NbAS timed)	Nb15 (SOS ALFA-NbALFA timed)	NbAS (not corrected, GFP ALFA-Nb ALFA timed)	NbAS (corrected, GFP ALFA-Nb ALFA timed)
k_{on} ($M^{-1}s^{-1}$)	BLI	$8.6 \pm 0.3 \times 10^4$	$8.6 \pm 0.3 \times 10^4$	$1.17 \pm 0.01 \times 10^5$	$1.17 \pm 0.01 \times 10^5$
	MS	$1.3 \pm 0.3 \times 10^5$	$1.2 \pm 0.4 \times 10^5$	$5 \pm 2 \times 10^4$	$4 \pm 3 \times 10^4$
k_{off} (s^{-1})	BLI	$1.41 \pm 0.01 \times 10^{-3}$	$1.41 \pm 0.01 \times 10^{-3}$	$1.163 \pm 0.003 \times 10^{-1}$	$1.163 \pm 0.003 \times 10^{-1}$
	MS	$1.3 \pm 0.2 \times 10^{-2}$	$5 \pm 3 \times 10^{-3}$	$1 \pm 2 \times 10^{-1}$	$2 \pm 3 \times 10^{-1}$
K_D	BLI	16.3±0.5 nM	16.3±0.5 nM	1.00±0.01 µM	1.00±0.01 µM
	MS	110±30 nM	40±20 nM	2±2 µM	3±3 µM

Table S3. MS parameters for GFP-EPEA binding to NbAS and Nb15 data collection as shown in **Figure 2**.

**Parmeter**	**Value**
Injection time	200 ms
In-source CID	0 eV
HCD	40 CE
Capillary temperature	200 °C
Source DC offset	10 V
Injection flatapole	9 V
Inter flatapole	6 V
Bent flatapole	4 V
Transfer multipole DC	2 V
C-trap entrance lens tune offset	0 V
Trapping gas	6 (UHV pressure ~$6x10^{-10}$ mbar)

Table S4. MS parameters for CA data collection as shown in **Figure 3A**.

**Parmeter**	**Value**
Injection time	200 ms
In-source CID	0 eV
HCD	10 CE
Capillary temperature	200 °C
Source DC offset	10 V
Injection flatapole	12 V
Inter flatapole	6 V
Bent flatapole	3 V
Transfer multipole DC	3.5 V
C-trap entrance lens tune offset	0 V
Trapping gas	2 (UHV pressure ~1.7×10^{-10} mbar)

Table S5. MS parameters for DHFR data collection as shown in **Figure 3B**.

**Parmeter**	**Value**
Injection time	200 ms
In-source CID	0 eV
HCD	10 CE
Capillary temperature	200 °C
Source DC offset	10 V
Injection flatapole	11 V
Inter flatapole	6 V
Bent flatapole	6 V
Transfer multipole DC	4.2 V
C-trap entrance lens tune offset	0 V
Trapping gas	2 (UHV pressure ~1.7×10^{-10} mbar)

Table S6. MS parameters for $KRas^{G12C}$ data collection as shown in **Figure 3C**.

**Parmeter**	**Value**
Injection time	200 ms
In-source CID	0 eV
HCD	10 CE
Capillary temperature	200 °C
Source DC offset	10 V
Injection flatapole	11.2 V
Inter flatapole	6 V
Bent flatapole	3 V
Transfer multipole DC	3.5 V
C-trap entrance lens tune offset	0 V
Trapping gas	2 (UHV pressure ~1.7×10^{-10} mbar)
